# Tricin selectively combats KRAS-mutant non-small cell lung cancer by inhibiting the PDGF-BB-induced SRC/MAPK/AP-1/PD-L1 signaling pathway and potentiating the antitumor effect of an anti-PD-1 antibody

**DOI:** 10.3389/fphar.2025.1594213

**Published:** 2025-06-17

**Authors:** Jia-Xin Li, Shi-Yu Tan, Li-Qi Li, Yu-Hong Zheng, Lin Zhao, Hui-Rong Zhu, Hai-Lang He, Yan-Yu Zhang, Run-Ze Li, Tian-Yu Bao, Yi-Zhong Zhang, Xiao-Man Yang, Hao Zhang, Hui-Hui Chen, Bo-Wen Wu, Xin Lin, Xiao-Sheng Lin, Yin Cheng Lin, Xin-Bing Sui, Ying Xie, Xian-Mei Zhou, Pei-Yu Yan

**Affiliations:** ^1^ Faculty of Chinese Medicine, State Key Laboratory of Quality Research in Chinese Medicines, Zhuhai MUST Science and Technology Research Institute, Macau University of Science and Technology, Macau, China; ^2^ Shuguang Hospital Affiliated to Shanghai University of Traditional Chinese Medicine, Shanghai, China; ^3^ Affiliated Hospital of Nanjing University of Chinese Medicine, Nanjing, China; ^4^ Shanghai University of Traditional Chinese Medicine, Shanghai, China; ^5^ Henan Key Laboratory of Traditional Chinese Medicine Prescription and Syndrome Signaling, Henan University of Chinese Medicine, Zhengzhou, China; ^6^ State Key Laboratory of Traditional Chinese Medicine Syndrome, Guangdong Provincial Academy of Chinese Medical Sciences, State Key Laboratory of Dampness Syndrome of Chinese Medicine, The Second Affiliated Hospital of Guangzhou University of Chinese Medicine (Guangdong Provincial Hospital of Chinese Medicine), Guangzhou, China; ^7^ Chinese Medicine and Translational Medicine R&D center, Zhuhai UM Science & Technology Research Institute, Zhuhai, China; ^8^ Guangzhou University of Chinese Medicine, Guangzhou, China; ^9^ BaoAn Central Hospital of Shenzhen, Shenzhen, China; ^10^ Faculty of Medicine, Macau University of Science and Technology, Macau, China; ^11^ School of Pharmacy, Hangzhou Normal University, Hangzhou, China

**Keywords:** tricin, KRAS, NSCLC, anti-PD-1 antibody, acute toxicity assay, network pharmacology, transcriptomics, SRC

## Abstract

**Background:**

KRAS is a commonly mutated gene that is present in approximately 30% of NSCLC patients. Currently, the identification of effective therapies for KRAS-mutant NSCLC is difficult for reasons of the structural and biochemical characteristics of the KRAS protein. Our previous study has revealed that tricin was a bioactive component having selective effects on KRAS^G12C^-mutant NSCLC cell lines. Thus, our aim in this project was to explore the mechanism by which tricin inhibited the progression of KRAS-mutant NSCLC much more deeply.

**Methods:**

First of all, we detected the acute toxicity of an intraperitoneal injection of tricin in mice according to the improved up-and-down procedure. Next, we integrated network pharmacology, molecular docking with transcriptomics analysis and biological methods to probe the underlying mechanisms of tricin in the treatment of patients with KRAS-mutant NSCLC. Furthermore, we explored the pharmaceutical effects of combination therapy with tricin and an anti-PD-1 inhibitor. Finally, we detected and analyzed the data from clinical samples to prepare for the clinical translation of tricin.

**Results:**

Intraperitoneal injection of tricin resulted in low acute toxicity. *In vitro*, tricin inhibited the migration, proliferation and colony formation of KRAS^G12C^-mutant NSCLC cells in a dose-dependent manner. Mechanistically, tricin inhibited KRAS^G12C^-mutant NSCLC cell growth primarily by suppressing the PDGF-BB-induced SRC/MAPK/AP-1/PD-L1 signaling pathway. SRC was identified as a potentially crucial target. *In vivo*, combined treatment with tricin and an anti-PD-1 antibody markedly suppressed the growth of tumors. The combination treatment had nearly no toxicity to the organs of the mice. In terms of immune regulation, tricin increased the numbers of CD8^+^ T lymphocytes and the levels of the functional cytokines TNFα, IFNγ, and Granzyme B. Tricin also increased the numbers of B lymphocytes and disrupted the PD-1/PD-L1 pathway. These results indicated that tricin could compensate for the deficiency of immunotherapy and enhance the antitumor activity of immunotherapy. Moreover, the detection of clinical samples indicated that the rate of SRC positivity was higher in elderly patients with KRAS mutations at the early stage. A positive correlation between the expression of SRC and PD-L1 was observed in tumor tissues.

**Conclusion:**

We believe that tricin is a safe and promising agent for the treatment of patients with KRAS-mutated NSCLC. Our study provides an experimental basis for improving the clinical application of traditional Chinese medicine.

## Graphical Abstract

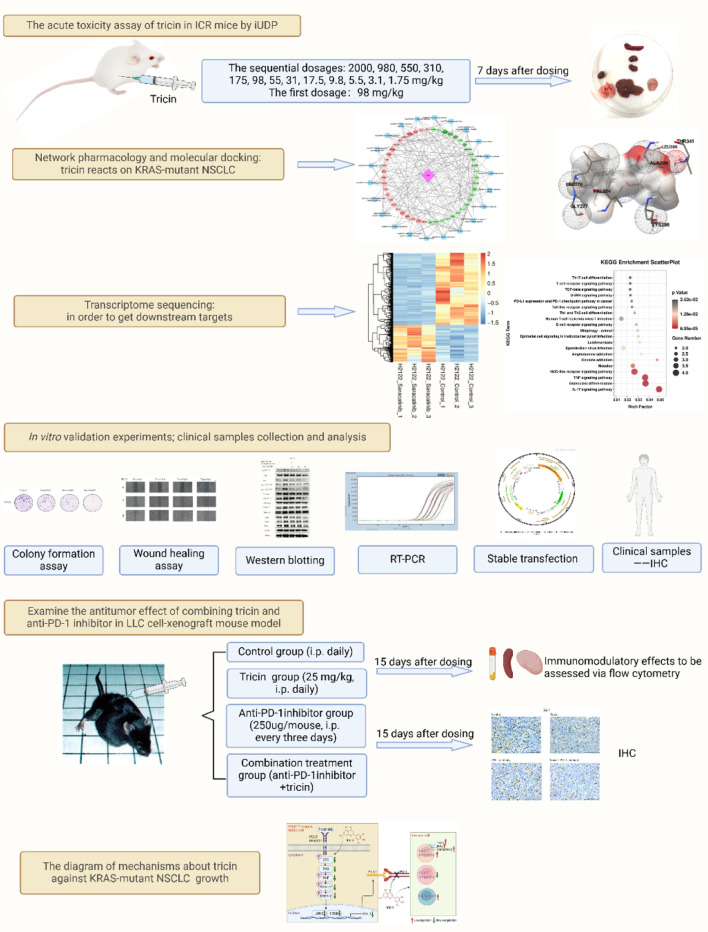

## Highlights

• For the first time, we integrated network pharmacology, molecular docking, and transcriptomic sequencing with biological methods to show that tricin inhibited KRAS^G12C^-mutant NSCLC cell growth mainly by dose-dependently suppressing the PDGF-BB-induced SRC/MAPK/AP-1/PD-L1 signaling pathway.
• For the first time, we found that combined treatment with tricin and an anti-PD-1 antibody markedly suppressed the growth of tumors and had nearly no toxicity to mouse organs. Tricin significantly increased the numbers of CD8^+^ T lymphocytes and B lymphocytes and regulated the PD-1/PD-L1 pathway, compensating for the deficiency of existing immunotherapy in clinical practice.
• For the first time, we explored the mechanism by which tricin inhibited the growth of KRAS-mutant NSCLC using multiple approaches, including analyses of cells, animals and clinical samples.

## 1 Introduction

Many long-term studies have revealed the characteristics of the genomic pathogenesis of tumors. The occurrence of cancer is closely related to cancer driver genes. This finding has the advantage of providing a therapeutic strategy called molecular targeted therapy. The Kirsten rat sarcoma viral oncogene (KRAS) is a commonly mutated gene that is expressed in approximately 30% of patients with non-small cell lung cancer (NSCLC). (Skoulidis and Heymach, 2019). Finding effective treatments to target KRAS-mutant NSCLC is notoriously difficult because of the structural and biochemical peculiarities of the KRAS protein. Currently, some actively recruiting and ongoing clinical trials regarding the treatment of KRAS-mutant NSCLC have received much attention. The novel covalent KRAS^G12C^ inhibitors sotorasib and adagasib have shown promising effects (Li et al., 2022). Approaches that inhibit KRAS-associated pathways also provide some benefit to patients. Immunotherapy is also in its infancy. However, acquired drug resistance still frequently occurs. Therefore, we hope to produce an increasing number of promising drugs to combat resistance.

Traditional Chinese herbal medicine is an important type of complementary and alternative medicine on account of its multiple targets, few adverse reactions, along with remarkable efficacy. In recent years, people have gained a deeper and more systematic understanding of Chinese herbal medicine treatment. Many studies have shown that Chinese herbal prescriptions and natural extracts are effective at treating tumors and assisting in the fight against drug resistance (Huang et al., 2022a). Tricin (formula: C_17_H_14_O_7_) is a naturally extracted flavonoid constituent found in some herbs such as *Rhizoma phragmitis* and some plants including bamboo, wheat, rice, maize, *Medicago sativa* and *Wikstroemia indica* (Lee et al., 1981; del Río et al., 2012; Lan et al., 2015; Wen et al., 2015; Miyamoto et al., 2019; Li J.-X. et al., 2021). Research conducted in the past 5 years has revealed that tricin has excellent pharmacological bioactivities. For instance, Professor Luo reported that tricin could be used to treat Parkinson’s disease by regulating autophagy (Wang et al., 2023). In addition, tricin was able to ameliorate acute colitis by suppressing colonic inflammation as well as regulating the gut microbiota profil (Li X.-X. et al., 2021). Moreover, various cancers and diseases have been effectively treated with tricin (Lee et al., 2020; Lee et al., 2022; Yue et al., 2020; Liu et al., 2022; Yang and Liu, 2022; Yang and Li, 2023). In our previous study, we reported that tricin is the main bioactive compound in *Rhizoma phragmitis* and that it exhibited selective cytotoxic effects on KRAS-mutant NSCLC cell lines such as H358, H2122 and LLC cells (Li J.-X. et al., 2021). Thus, the mechanism by which tricin inhibits the progression of KRAS-mutant NSCLC should be explored in greater depth.

The gradual evolution of the practice of traditional herbal medicine into an evidence-based medical system requires the support of modern technology and is not exclusively based on the experience of the physicians themselves. In the current research on traditional Chinese medicine, new technologies, such as network pharmacology, molecular docking, metabolomics and transcriptomics, are commonly used to study the mechanisms involved. Some more advanced omics technologies, such as spatial transcriptomic and spatial proteomic technologies, are still rare in the field of traditional Chinese medicine and need more attention in the future. The combination of multiomic techniques and bioinformatics can help researchers explore the mechanism of Chinese herbal medicine from various angles and aspects, which is helpful for promoting the wide application of Chinese herbal medicine in clinical practice and is highly important for promoting the modernization of traditional Chinese medicine.

Herein, for the first time, we have integrated a bioinformatic analysis with a transcriptomic analysis and biological methods to understand the underlying mechanisms by which tricin suppresses the progression of KRAS-mutant NSCLC. First and foremost, we tested the acute toxic effects of an intraperitoneal injection of tricin in ICR female mice via an improved up-and-down procedure. Next, we investigated the underlying mechanism to develop a new natural extract, tricin, for treating patients with KRAS-mutant NSCLC who are currently clinically refractory to treatment. Furthermore, we explored the pharmaceutical effect of a combination therapy with tricin and an anti-PD-1 inhibitor, providing new ideas for research on combination treatment for patients with KRAS-mutant NSCLC. Last but not least, we detected and analyzed the data from the clinical samples to prepare for clinical translation. Our study showed that the naturally extracted compound tricin is a promising candidate treatment for patients.

## 2 Materials and methods

### 2.1 Reagents and cell culture

H358, H2122 and LLC cells were purchased from the National Collection of Authenticated Cell Cultures (Shanghai, China). The cells were cultured in RPMI 1640 medium or DMEM medium supplemented with 10% FBS and 1% PS (penicillin‒streptomycin solution). The main reagents relevant to the experiments were listed in Supplementary Table S1.

### 2.2 Acute toxicity assay

Female ICR mice were used in this study. This animal experiment was approved by the Animal Ethics Committee of Macau University of Science and Technology (Registration number: AL019/DICV/DIS/2019). According to the results of previous studies, oral tricin has very low toxicity. Therefore, the initial estimated LD_50_ was set to 175 mg/kg, the slope was set to 4, and the sigma value was 0.25. At this time, the experimental dose gradients provided by AOT425StatPgm software were as follows: 2,000, 980, 550, 310, 175, 98, 55, 31, 17.5, 9.8, 5.5, 3.1, and 1.75 mg/kg. A concentration of 98 mg/kg was administered as the first dose to the first ICR mouse. The survival and death of the mouse within 24 h were recorded. The next day, if the first mouse died (denoted as X), the second mouse was administered tricin at a dose of 55 mg/kg. If the first mouse was alive (denoted as O), a dose of 175 mg/kg was selected. This process was performed sequentially at the doses calculated by the software until any of the following stopping criteria appeared:

(1) Three consecutive mice survived at the highest dose of 2000 mg/kg, namely, OOO;
(2) Five survival and death flips occurred in six consecutively treated mice, namely:

**Table:** 

OXOXOX		XOXOXO
ΟOΧΟΧΟΧΟ		XXOXOXO
OΟOΧΟΧΟΧΟ		XXXOXOXO
OΟOOΧΟΧΟΧΟ		XXXXOXOXO
OOΟOOΧΟΧΟΧΟ	OR	XXXXXOXOXO
OOOΟOOΧΟΧΟΧΟ		XXXXXXOXOXO
OOOOΟOOΧΟΧΟΧΟ		XXXXXXXOXOXO
OOOOOΟOOΧΟΧΟΧΟ		XXXXXXXXOXOXO
OOOOOOΟOOΧΟΧΟΧΟ		XXXXXXXXXOXOXOX

The surviving mice were observed for 7 days, and body weights were recorded every other day. At the end of the 7-day observation, each mouse was sacrificed to end the experiment. The animals were dissected for pathological observations. The body weight and weights of brain, heart, liver, spleen, lung and kidney were measured, and the organ indices were calculated. LD_50_ values were calculated according to the methods of Professor Zhou’s research team and with AOT425StatPgm software (Zhang et al., 2022).

### 2.3 Network pharmacology analysis

The BATMAN, TCMSP, and SwissTargetPrediction databases and the literature were separately searched to predict the corresponding protein targets of tricin. The predicted targets were subsequently merged. Next, the USCC Xena database was used to download standardized RNA-seq data from patients with lung adenocarcinoma in TCGA. Information on the KRAS-mutant samples was obtained using the cBioPortal tool. TCGA LUAD samples were divided into a KRAS-mutant NSCLC group (Mut) and a pericarcinomatous tissue group (NC). A differential analysis of the Mut and NC groups was then performed using the limma package in the R language. Notably, |logFC| > 0.5 and FDR <0.05 were chosen as thresholds for significant differences. Tricin-related targets and targets of KRAS-mutant NSCLC were imported into the VENNY 2.1 online mapping tool to obtain drug‒disease intersection targets and produce the corresponding Venn diagrams. GO function and KEGG pathway enrichment analyses of the overlapping targets were performed using the DAVID tool. The STRING v11.5 database was used to analyze the protein‒protein interactions (PPIs) of the overlapping targets. A required confidence (combined score) > 0.4 was selected as the threshold for the PPI. Then, Cytoscape software was used to construct the network diagram. The top twenty targets of KRAS-mutant NSCLC treated with tricin were screened by referring to degree values. Finally, a pharmacological network was constructed using Cytoscape software to identify the key targets and related signaling pathways affected by tricin in the treatment of KRAS-mutant NSCLC.

### 2.4 Molecular docking analysis

The 3D molecular structure of tricin was downloaded from the PubChem Compound database. PDB format files of macromolecular proteins were downloaded from the PDB database. Operations such as water removal, ligand molecule removal, and residue removal were performed with tools such as PyMOL and ADT for subsequent analysis. AutoDock Vina software was used to study the docking between the target protein and molecules. The Lamarckian genetic algorithm (LGA) was used as the docking algorithm. The lower the parameter is, the higher the affinity is. Local binding sites were analyzed using AutoDock.

### 2.5 Colony formation assay

Approximately five hundred cells were planted in each well. After overnight culture, the cells were treated with different concentrations of tricin for approximately 14 days. Importantly, the medium was changed every 3 days. When cell colony formation was obvious, the cells were washed with PBS and fixed with 4% paraformaldehyde for approximately 30 min at 4°C. The cells were then stained with a crystal violet solution for more than 30 min. The samples were subsequently rinsed to remove the excess crystal violet solution, dried, and the colonies were photographed.

### 2.6 Wound healing assay

The cells were planted and allowed to grow to approximately 90% confluence. The cell monolayers were scratched with 10 μL pipette tips. The cell debris was then removed by washes with PBS. The cells were treated with the indicated concentrations of tricin for 0 h, 12 h and 24 h, and the intercellular space images were captured under a microscope. At the same time, the gap widths were estimated using ImageJ software.

### 2.7 Real-time PCR assay

Approximately 2*10^5^ to 3*10^5^ cells were seeded in each well. The cells were treated with various concentrations of tricin for 24 h. Total RNA was extracted from the cells using Trizol reagent. Then, DNA was obtained with a reverse transcription kit manufactured by TOYOBO. Next, FastStart Universal SYBR Green Master was used to conduct the real-time PCR assay. The mixtures were incubated at 50°C for 2 min and 95°C for 10 min. The samples were circulated forty cycles of the procedure, which was set at 95°C for 15 s, 60°C for 1 min, then 95°C for 15 s, 60°C for 1 min, 95°C for 15 s. GAPDH was used as the reference gene. Gene expression levels were calculated using the 2^-△△Ct^ method. Supplementary Table S2 showed the sequences of the PCR primers used.

### 2.8 Transcriptomic sequencing

Total RNA was extracted from the samples from six groups (H358 control group; H358 saracatinib group; H2122 control group; H2122 saracatinib group; LLC control group; and LLC saracatinib group) using Trizol reagent. Illumina sequencing was conducted by LC-Bio Technology Co., Ltd. The amplification conditions included initial denaturation at 95°C for 3 min; eight cycles of denaturation at 98°C for 15 s, annealing at 60°C for 15 s, and elongation at 72°C for 30 s; and a final extension at 72°C for 5 min. Bioinformatics analyses consisted of sequencing, filtering clean reads, alignment with the reference genome, quantification of gene abundance, an analysis of differentially expressed genes (DEGs), analysis of the relationships between samples, GO enrichment analysis, and KEGG pathway enrichment analysis. DESeq2 software was used to analyze the differentially expressed genes between two groups. For this experiment, |log_2_FC|≥1 and q < 0.05 were used as the thresholds (FC represents the fold change). KEGG pathway enrichment analyses of the differentially expressed genes were performed.

### 2.9 Western blot assay

Approximately 2*10^5^ to 3*10^5^ cells were seeded in each well to extract proteins. The cells were treated with various concentrations of tricin for 24 h and then treated with PDGF-BB mouse (rmPDGF-BB) or PDGF-BB human (rhPDGF-BB). The total protein concentration was detected with a Pierce™ BCA protein assay kit. Next, lysates were separated on 10% SDS-acrylamide gels in our experiments. The operating voltage was set at 80 V for 30 min, and then it was set at 140 V for approximately 1 h. Proteins were transferred to a membrane at 300 mA for 2 h (high-molecular-weight proteins) or at 250 mA for 50 min (low-molecular-weight proteins). The membrane was then blocked in 5% BSA for 1 h at room temperature on a shaker. The membrane was incubated overnight with relevant primary antibodies (p-SRC^Y416^, T-SRC, Ras, p-C-Raf^Ser338^, p-C-Raf^Tyr340^, T-C-Raf, p-MEK1/2, T-MEK1/2, p-ERK1/2, T-ERK1/2, JUNB, FOSB, PD-L1, DUSP2 and GAPDH rabbit antibodies) on a shaking table at 4°C. The next day, the membrane was incubated with fluorescein-labeled rabbit antibodies for 2 h on a shaking table in the dark. The intensity of each band was detected using a LI-COR Odyssey imaging scanner.

### 2.10 Stable transfection assay

The SRC-overexpressing lentivirus was constructed with the pLV-CMV-MCS-EF1-ZsGreen1-T2A-Puro vector. The fluorescent label used was ZsGreen1. H358, H2122 and LLC cells were transfected with the SRC-overexpressing lentivirus or the control lentivirus. The transfected cells were subjected to Western blot, wound healing, and colony formation assays.

### 2.11 Mouse xenograft assay

Six-to eight-week-old female C57BL/6J mice were used for the study. This animal experiment was approved by the Animal Ethics Committee of Macau University of Science and Technology (Registration number: AL019/DICV/DIS/2019, Date: 12/11/2019). The experiment was conducted according to the method of modeling and drug dosages established in a previous study (Li J.-X. et al., 2021). When the tumors reached approximately 50–100 mm^3^ in size, the mice were randomly divided into four groups: the control group (i.p. daily), tricin group (25 mg/kg, i.p. daily), anti-PD-1 inhibitor group (250 μg/mouse, i.p. every 3 days) and combination group. Tumor volumes and body weights were measured every 3 days in each group of mice. The mice were sacrificed after 15 days, and the tumor weights were measured. Spleens, tumors and plasma samples were collected for subsequent IHC staining and flow cytometry analysis. The organ index was calculated with the following formula: organ index = organ weight (mg)/mouse weight (g).

### 2.12 Evaluation of immune responses by flow cytometry

An experiment was performed to detect indices of the immune response on the surface of blood, spleen, and tumor cells. After the animal experiments, whole blood from the eyeball or a suspension of the spleen or tumor obtained after grinding was centrifuged at 3,000 rpm for 10 min at 4°C. The supernatant was removed, and the remaining sediments contained blood cells, spleen cells or tumor cells. Then, antibodies against CD3, CD45, CD4, CD8, CD19, NK1.1 and PD-1 were added to each sample. After 30 min of staining at 4°C in the dark, the cells were centrifuged again. Red blood cell lysis buffer was added to each sample, and the samples were vortexed for several seconds. The samples were allowed to stand for 10 min. When the solution became clear, it was centrifuged at 3,000 rpm for 3 min at 4°C. The supernatant was removed, and the remaining cells in the sheath fluid were detected using a BECKMAN CytoFLEX flow cytometer.

The other experiment involved the detection of intracellular indices of the immune response in blood, spleen, and tumor cells. Whole blood from the eyeball or a suspension of the spleen or tumor obtained after grinding was centrifuged at 3,000 rpm for 10 min at 4°C. The supernatant was removed. Red blood cell lysis buffer was added to each sample, and the samples were vortexed for several seconds. The samples were allowed to stand for 10 min. When the solution became clear, it was centrifuged at 3,000 rpm for 3 min at 4°C. The supernatant was removed, and the remaining sediment contained white blood cells. Then, RPMI 1640 medium and the cell activation cocktail (2 μL of the cell activation cocktail per 1 mL of RPMI 1640 medium) were added to white blood cells. The cells were incubated in the incubator for 4–6 h, after which they were flicked every hour. Antibodies against CD45, CD4 and CD8 were added to each sample. After 30 min of staining at 4°C in the dark, the cells were centrifuged. The Fixation and Permeabilization Buffer Set (ratio of Fixation/Permeabilization Concentrate to Fixation and Permeabilization Diluent = 1:3) was added to each sample. After 30 min of staining at 4°C in the dark, the cells were centrifuged again. Next, antibodies against TNF-a, IFN-γ and Granzyme B were added to the permeabilization buffer separately and mixed, and the mixture was added to each sample. After 30 min of staining at 4°C in the dark, the cells were centrifuged. The supernatant was removed, and the remaining cells in the sheath fluid were detected using a BECKMAN CytoFLEX flow cytometer.

Using beads firstly to conduct the compensation experiments were required. All the data were analyzed using the software provided with the instrument.

### 2.13 Immunohistochemistry assay

Tumor samples from the mice were fixed overnight with 4% PFA and dehydrated overnight in 80% ethyl alcohol. The samples were embedded in paraffin and then sliced for immunohistochemical staining. First, the slices were sequentially incubated with xylene I, xylene II, xylene III, absolute ethanol I, absolute ethanol II, 85% alcohol, and 75% alcohol, after which they were rinsed with distilled water. Next, the tissue slices were subjected to antigen retrieval. They were heated at medium power until the solution boiled and then transferred to medium–low power. The slices were subsequently placed in PBS and shaken on a shaker three times. The slices were subsequently placed in 3% hydrogen peroxide and incubated at room temperature in the dark for approximately twenty-five minutes. The slices were placed in PBS and shaken three times again. Then, 3% BSA was added to cover the tissue, and the tissues were sealed for approximately 30 min at room temperature. Thirty minutes later, each primary antibody was added to the slices, and the slices were incubated overnight at 4°C. The next day, the slices were placed in PBS and washed three times. The tumor tissues were covered with secondary antibodies and incubated at room temperature for approximately 1 h. Next, the slices were placed in PBS and washed three times again. DAB color development solution was added to the slices. The positive samples were brownish yellow. The slices were rinsed with tap water to terminate the reaction. Furthermore, the slices were counterstained with hematoxylin staining solution, washed with tap water, and differentiated with hematoxylin differentiation solution. Then, the slices were washed with tap water, treated with a hematoxylin bluing solution and then washed with running water. Finally, the slices were sequentially placed in 75% alcohol, 85% alcohol, absolute ethanol Ⅰ, absolute ethanol Ⅱ, N-butanol, and xylene Ⅰ. The slices were removed from xylene and dried slightly. The slices were mounted with neutral gum. Images were captured using a Nikon Eclipse E600 microscope (×400 magnification, scale bar = 100 µm).

### 2.14 Collection and analysis of clinical samples

Tumor samples from 24 patients with KRAS-mutant NSCLC diagnosed were collected between 2019.11 and 2023.12. This retrospective study was given permission to conduct by the ethics committee of Jiangsu Province Hospital of Chinese Medicine (Approval Number: 2024NL-234-02). Using the records from the electronic medical system, all patient characteristics, including sex, age, smoking history, time of diagnosis, degree of tumor cell differentiation and tumor node metastasis (TNM) stage, were obtained. Immunohistochemical analyses were performed by TEKSQRAY (Shenzhen, China). The results were scored by professional pathologists based on the proportion of positively stained cells along with the staining intensity. The proportion of positively stained tumor cells was classified as follows: 0 (<5%), 1 (6%–25%), 2 (25%–50%), 3 (50%–75%), and 4 (>75%). Moreover, the intensity was classified as follows: 0 (no staining), 1 (weak staining), 2 (moderate staining) or 3 (strong staining). The two results were summed to obtain the IHC score.

### 2.15 Statistical analysis

All data were the mean and standard error of three independent experiments and were presented as mean ± SEM. ImageJ software was used for quantitative analyses of the wound healing, immunohistochemistry and Western blot data. GraphPad Prism 9.5.1 software was used for graph generation and statistical analyses. The relationships between the expression of SRC, JUNB, FOSB and PD-L1 and clinicopathological features were analyzed via chi-square χ^2^ tests using IBM SPSS Statistics 21. The correlations between the expression of SRC, JUNB, FOSB, and PD-L1 were also analyzed via chi-square χ^2^ tests using IBM SPSS Statistics 21. A t-test (comparing the differences in the means between two groups) and one-way ANOVA (comparing the differences in the means between three or more groups) were used to analyze the data. *P values <0.05, **P values <0.01 and ***P values <0.001 indicate statistically significant differences.

## 3 Results

### 3.1 An improved up-and-down procedure was used to examine the acute toxicity of an intraperitoneal injection of tricin in ICR mice

**FIGURE 1:**
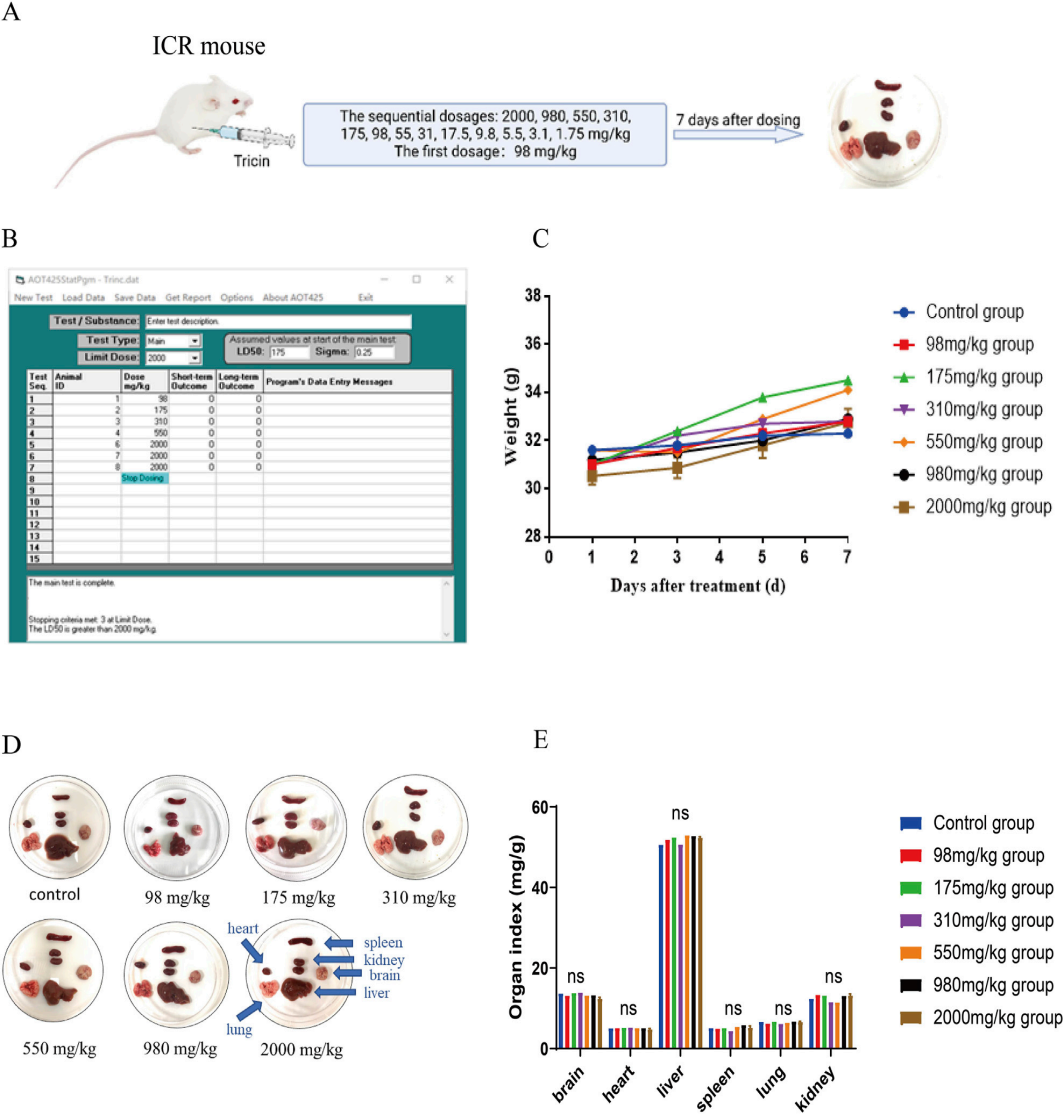
An improved up-and-down procedure was used to examine the acute toxicity of an intraperitoneal injection of tricin in ICR mice. **(A)** The brief workflow of this experiment was exhibited. The surviving mice were observed for 7 days. At the end of the 7-day observation, each mouse was sacrificed to end the experiment. They were dissected for pathological observation. **(B)** The operating interface of the software AOT425StatPgm was displayed. The AOT425StatPgm software could directly calculate the LD_50_ value. **(C)** Body weights of the surviving mice in each group were recorded every other day until the seventh day after treatment. **(D)** The photos of the brain, heart, liver, spleen, lung and kidneys of mice administered with different concentrations of tricin and the normal group were shown. **(E)** The bar graph about organ indexes of female ICR mice treated with different dosages of tricin by intraperitoneal injection. ns: not significant vs. Control group.

An acute toxicity test is essential for the development of a drug. Our previous research revealed that an intraperitoneal injection of tricin significantly inhibited the growth of lung cancer cells *in vivo* (Li J.-X. et al., 2021). Currently, no acute toxicity result has been reported for the intraperitoneal injection of tricin. Therefore, examining the acute toxicity of an intraperitoneal injection of tricin in ICR mice is important. The improved up-and-down method shortens the experimental period and improves the effect. In addition, it is useful for detecting the toxicity of natural products with low yields or high prices. Figure 1A exhibited the brief workflow of this experiment. In accordance with the improved method, the dosage of treatment and survival of the ICR mice after 24 h were recorded using AOT425StatPgm software. The AOT425StatPgm software directly calculated an LD_50_ value for the intraperitoneal injection of tricin greater than 2,000 mg/kg (Figure 1B). During the experiment, the body weights of the surviving mice were recorded every other day. We found that the body weights of the mice in each group steadily increased each day, indicating that tricin had little effect on the body weights of the mice in the short term (Figure 1C). At the end of the 7-day observation, the mice all exhibited a good mental state and were active. Figure 1D showed photos of the organs of mice treated with different concentrations of tricin, including the brain, heart, liver, spleen, lung and kidney. When a high concentration of tricin (higher than 175 mg/kg) was injected intraperitoneally into mice, the solubility of tricin was not very good, and residual yellow tricin was observed on the surface of the liver. No significant change was observed in the other organs. Figure 1E displayed that tricin had nearly no significant effect on organ indices compared with those of normal mice. The classification criteria for acute toxicity can be divided into five categories depending on the LD_50_ value (Nations, 2011). In summary, the effects of the intraperitoneal injection of tricin can be classified into Category V (low acute toxicity).

### 3.2 Integrating network pharmacology with molecular docking analysis forecasted the possible target-pathway interaction about the cancer inhibitory influence of tricin on KRAS-mutant NSCLC

**FIGURE 2:**
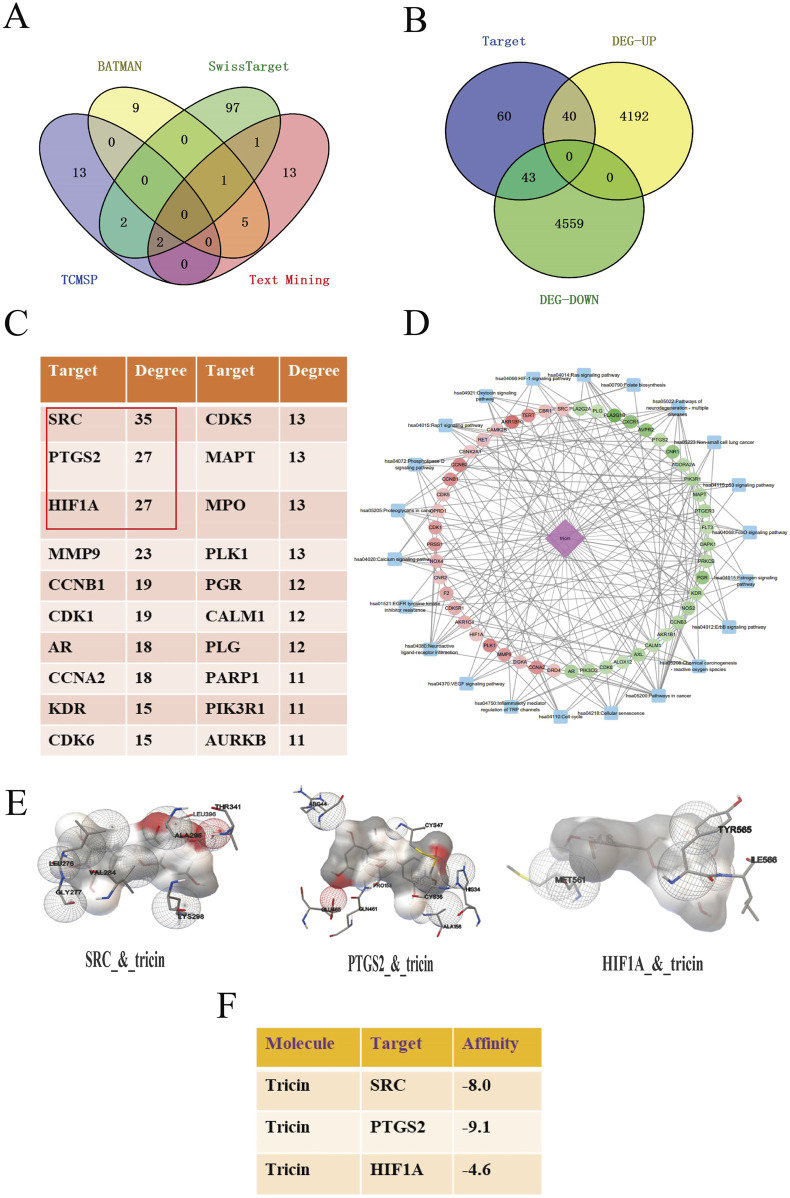
Integrating network pharmacology with molecular docking analysis forecasted the possible target-pathway interaction about the cancer inhibitory influence of tricin on KRAS-mutant NSCLC. **(A)** Protein targets in tricin were obtained by a combined search of three databases and literatures. **(B)** Venn diagram about overlapping targets between tricin and KRAS-mutant NSCLC was exhibited. **(C)** The hub targets of tricin anti-KRAS mutated NSCLC were predicted by network topologic analysis, and the top 20 targets were shown. **(D)** The component-target-pathway network was constructed. Pink diamond represented the small molecule component, dots represented hub targets, and blue squares represented cancer-related pathways. **(E)** The local maps of the best model visualizations about tricin docking core targets SRC, PTGS2 and HIF1A were displayed. **(F)** The docking scores of tricin and core targets were calculated. Tricin had scores less than −5 with both SRC and PTGS2, indicating strong binding activity.

Given that tricin is a naturally extracted compound with low toxicity, its potential for clinical application is clear. Based on the results of our previous study, the mechanism by which tricin inhibits the progression of KRAS-mutant NSCLC should be explored in greater depth. The integration of network pharmacology with molecular docking analysis can be used to predict possible compound‒target‒pathway interactions. First, a total of 143 protein targets of tricin were obtained through a combined search of three databases and the literature, as shown in Figure 2A. A total of 154 samples were included in the KRAS-mutant NSCLC group (Mut) and 59 samples in the pericarcinomatous tissue group (NC) from TCGA (The Cancer Genome Atlas). A total of 8,834 differentially expressed genes were identified in KRAS-mutant NSCLC patients. These 8,834 disease targets intersected with 143 protein targets of tricin, and 83 intersecting targets were identified (Figure 2B). Supplementary Figure S1 exhibited the top 20 biological processes and KEGG enrichment pathways of the overlapping targets ranked by significance. The key biological processes included protein phosphorylation, negative regulation of gene expression, response to drugs, the inflammatory response, the carbon metabolic process and so on. Moreover, the enriched KEGG pathways included metabolic pathways, pathways related to cancer, nitrogen metabolism, inflammatory mediator regulation of TRP channels, and EGFR tyrosine kinase inhibitor resistance, suggesting that tricin was extensively involved in pharmacological regulation. At the same time, the PPI relationship of the 83 overlapping targets was predicted and the hub genes in the PPI network were scored via a network topology analysis. The top 20 targets were shown in Figure 2C. Finally, the hub targets and cancer-related pathways were selected to construct a pharmacological network, as shown in Figure 2D and Supplementary Table S3. The results revealed that SRC, PTGS2 and HIF1A were considered the most critical hub targets of tricin in the treatment of KRAS-mutant NSCLC. These hub targets were closely associated with cancer-related pathways.

Furthermore, molecular docking was performed to assess the affinity between tricin and key targets. The PDB ID for the secondary structure (PDB ID) of SRC was 7NG7, PTGS2 was 3TZI, and HIF1A was 5L9B. The affinity of tricin for these three targets was assessed using a docking algorithm called LGA (Morris et al., 1998). It is generally believed that the affinity score of less than −5 indicates that the docking result is more reliable, and the smaller the score is, the greater the affinity is. Figure 2E exhibited the local maps of the best model visualizations. Figure 2F showed that tricin had affinity scores less than −5 with both SRC and PTGS2, displaying strong binding activity.

### 3.3 Tricin inhibited the migration, proliferation and colony formation of KRAS^G12C^-mutant NSCLC cells in a dose-dependent manner

**FIGURE 3:**
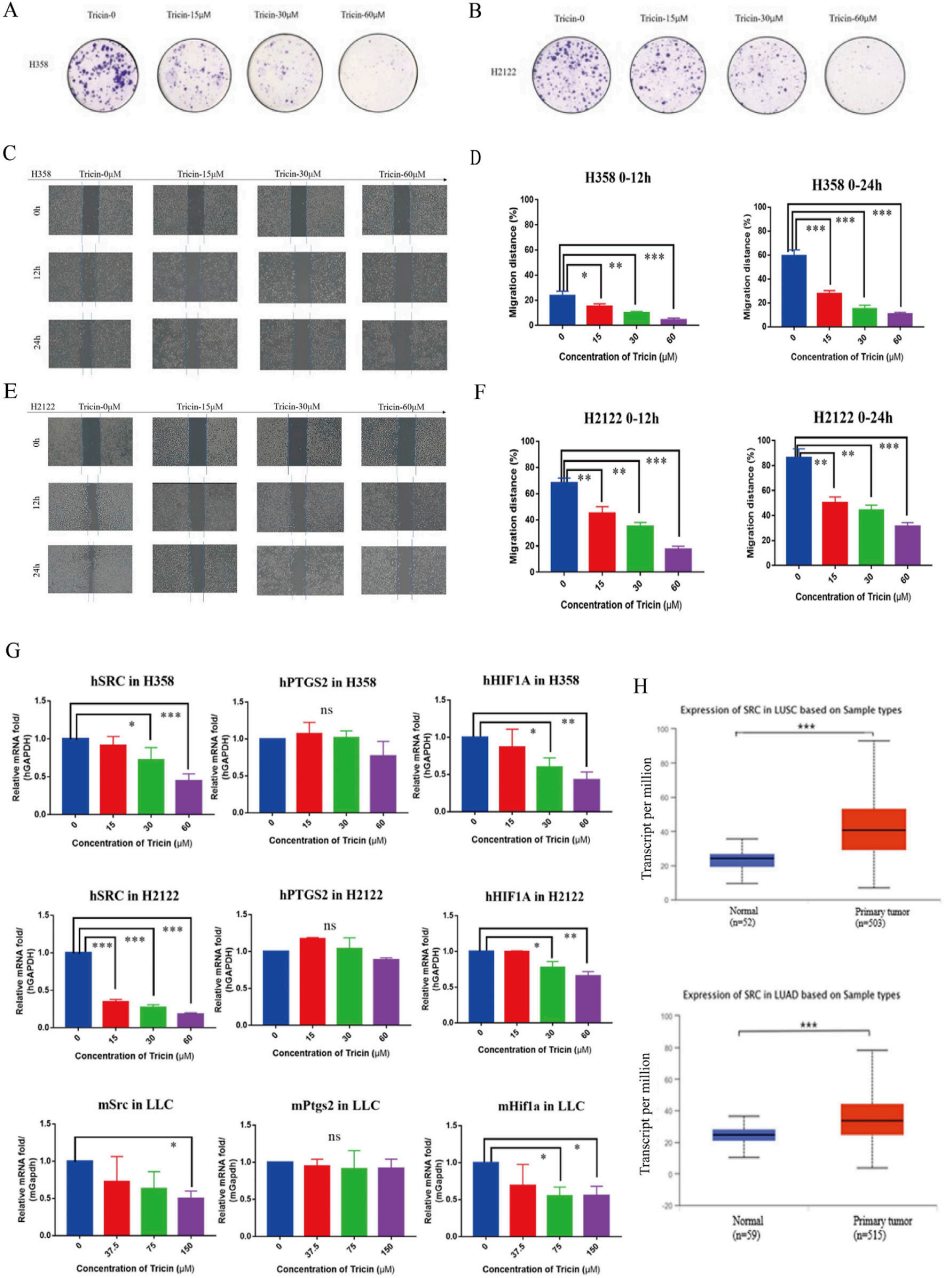
Tricin inhibited the migration, proliferation and colony formation of KRAS^G12C^-mutant NSCLC cells in a dose-dependent manner. Meanwhile, tricin significantly reduced the mRNA expression level of SRC gene in KRAS^G12C^-mutant NSCLC cells. **(A,B)** The colony formation of H358 and H2122 cells treated with different concentrations of tricin were shown. **(C–F)** The cell migration of H358 and H2122 cells treated with different concentrations of tricin at 12 h and 24 h were exhibited. This experiment was repeated three times. Then the gap widths were calculated and the data were analyzed. All data were presented as mean ± SEM (*P < 0.05, **P < 0.01, ***P < 0.001). **(G)** RT-PCR assay was conducted to determine the expression of SRC, PTGS2 along with HIF1A on H358, H2122 and LLC cells after the 24 h tricin treatment. All data were presented as mean ± SEM (*P < 0.05, **P < 0.01, ***P < 0.001). **(H)** The expression of proto-oncogene SRC was compared between tumor tissues and normal lung tissues in LUSC and LUAD patients collected from TCGA. All data were presented as mean ± SEM (***P < 0.001).

Our preliminary study indicated that tricin had selective and dose-dependent cytotoxic effects on the KRAS^G12C^-mutant H358, H2122 and LLC cells. The IC_50_ values of tricin in H358, H2122 and LLC cells after 72 h of treatment were 30.78 ± 1.21 μM, 38.46 ± 1.12 μM and 77.98 ± 1.21 μM, respectively (Li J.-X. et al., 2021). Meanwhile, we have verified that tricin could inhibit proliferation, migration as well as the colony formation of LLC cells in a dose-dependent manner (Li J.-X. et al., 2021). So as to determine if tricin has these effect on human KRAS^G12C^-mutant NSCLC cells, we examined H358 and H2122 cells. Supplementary Figure S2 showed that the number of surviving H358 and H2122 cells decreased significantly after 24 h of treatment with increasing concentrations of tricin. In addition, tricin obviously inhibited the colony-forming ability of H358 and H2122 cells in a dose-dependent manner (Figures 3A,B). Moreover, as shown in Figures 3C,E, the gaps of the scratch wounds of the cells were comparable at 0 h. However, tricin significantly and dose-dependently slowed the migration of H358 and H2122 cells after 12 h and 24 h. The gap widths were calculated, and the data were analyzed, which revealed that tricin significantly inhibited the migration of H358 and H2122 cells (Figures 3D,F).

### 3.4 Tricin significantly reduced the mRNA expression level of SRC gene in KRAS^G12C^-mutant NSCLC cells

Network pharmacology analysis has been used to predict that tricin might play vital roles in inhibiting KRAS-mutated NSCLC by targeting hub genes including SRC, PTGS2 and HIF1A. Meanwhile, results of molecular docking exhibited tricin had strong binding activity with SRC and PTGS2. Therefore, we conducted a variety of molecular experiments to verify the therapeutic mechanism by which tricin inhibited the proliferation of the KRAS^G12C^-mutant NSCLC cell lines H358, H2122 and LLC. As shown in Figure 3G, the quantitative PCR results revealed that the expression of the gene encoding HIF1A decreased following treatment with different concentrations of tricin, and the expression of the gene encoding SRC decreased most significantly, while the expression of the gene encoding PTGS2 showed no obvious trend. Combining the results of network pharmacology, molecular docking and RT‒PCR assays, the SRC gene was ultimately selected as the main gene for our study.

We detected SRC expression in the human lung squamous cell carcinoma (LUSC) and human lung adenocarcinoma (LUAD) transcriptome datasets from The Cancer Genome Atlas (TCGA) to determine whether our findings have clinical implications. The expression of proto-oncogene SRC was significantly increased in tumor tissues compared with normal lung tissues (Figure 3H).

### 3.5 Transcriptome sequencing predicted that the common downstream targets of SRC in KRAS^G12C^-mutant NSCLC cells were JUNB, FOSB and DUSP2, which were related to inflammatory and immune pathways

**FIGURE 4:**
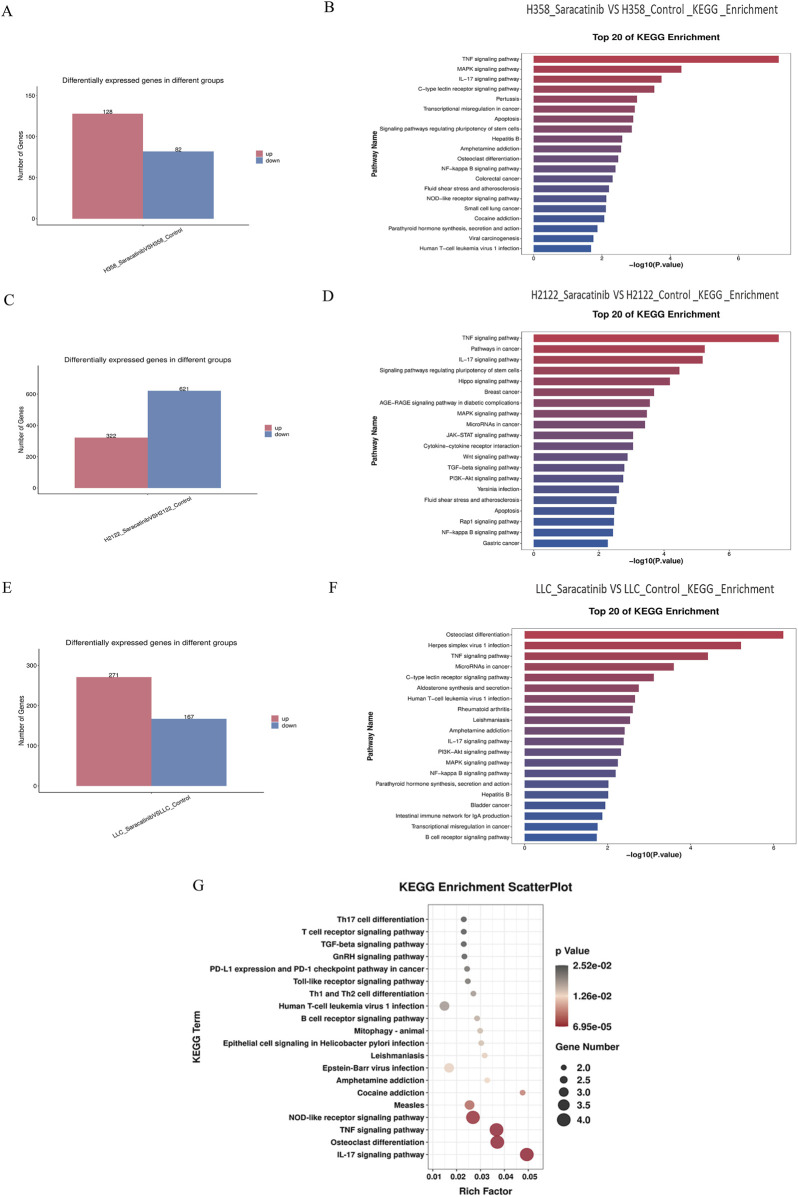
Transcriptome sequencing predicted that the common downstream targets of SRC in KRAS^G12C^-mutant NSCLC cells were JUNB, FOSB and DUSP2, which were related to inflammatory and immune pathways. **(A,C,E)** Bar graphs about the differential gene expressions between saracatinib group and control group in H358, H2122 and LLC cells were exhibited. **(B,D,F)** The top 20 of KEGG pathways about differentially expressed genes between saracatinib group and control group in H358, H2122 and LLC cells were displayed. TNF signaling pathway, IL-17 signaling pathway, MAPK signaling pathway and NF-κB signaling pathway were common signaling pathways to all three cell lines. **(G)** The KEGG enrichment scatterplot in connection with all common differential genes in H358, H2122 and LLC cells were shown. These pathways were mainly concentrated in the direction of inflammation and immunity, including the PD1/PD-L1 pathway.

Cells treated with the potent SRC inhibitor saracatinib and cells in the control group were subjected to transcriptome sequencing for the sake of understanding the downstream genes and pathways of SRC in KRAS-mutant NSCLC cells. First and foremost, based on the results of the MTT and RT‒PCR assays, we concluded that the most suitable concentrations of saracatinib for H358, H2122 and LLC cells were 5 μM for 2 h, 1.25 μM for 3.5 h and 2.5 μM for 1.5 h, respectively. Next, total RNA was extracted from 18 samples from six groups (H358 control group; H358 saracatinib group; H2122 control group; H2122 saracatinib group; LLC control group; and LLC saracatinib group) using Trizol reagent. Sequencing and data analyses were performed with the help of LC-Bio Technology Co., Ltd. The differentially expressed genes between two groups were shown in bar graphs (Figures 4A,C,E). Notably, |log_2_FC|≥1 and q < 0.05 were used as the thresholds (FC represents the fold change). KEGG pathway enrichment analyses of the differentially expressed genes were performed. Figures 4B,D,F showed the top 20 KEGG pathways for each cell line. The TNF signaling pathway, the IL-17 signaling pathway, the MAPK signaling pathway and the NF-κB signaling pathway were common signaling pathways identified in all three cell lines. Table 1 summarized the differentially expressed genes associated with these four common pathways in the three cell lines, among which JUNB, FOSB and DUSP2 were the differentially expressed genes shared by the three cell lines. Furthermore, Figure 4G showed the KEGG pathways related to all the common differentially expressed genes identified in the three cell lines in a bubble scatterplot. We found that these pathways, including the PD-1/PD-L1 pathway, were involved mainly in inflammation and immunity.

**TABLE 1:** The differential genes in connection with four common pathways in H358, H2122 and LLC cells.

	TNF signaling pathway	IL-17 signaling pathway	MAPK signaling pathway	NF-κB signaling pathway
H358	JUN, NFKBIA, TNFAIP3, JUNB, LIF, IRF1, FOS, CREB5, BIRC3, CXCL5	JUN, NFKBIA, TNFAIP3, FOSB, FOS, CXCL5	JUN, DUSP5, GADD45B, NR4A1, SRF, GADD45A, FOS, DUSP1, DUSP8, DUSP2, NGFR	NFKBIA, TNFAIP3, GADD45B, GADD45A, BIRC3
H2122	EDN1, MMP3, IL1B, CXCL2, NOD2, JAG1, CXCL1, CXCL3, JUN, BCL3, JUNB, TNFRSF1A, IL15, PIK3CD, NFKBIA, CEBPB, PTGS2, CASP10, TNFAIP3	MAPK15, MMP3, IL1B, CXCL8, CXCL2, CXCL1, CXCL3, CSF3, JUN, NFKBIA, CEBPB, PTGS2, TNFAIP3, FOSB	DUSP2, MYC, KITLG, KIT, IL1B, DUSP6, IL1A, JUN, DUSP10, TNFRSF1A, VEGFA, FGF19, RPS6KA1, PLA2G4F, RAC2, RELB, NFATC1, EFNA3, FGF5, MKNK2, EFNA4, FLT3LG, RASGRP1	IL1B, CXCL8, CXCL2, CXCL1, CXCL3, TNFRSF1A, NFKBIA, PTGS2, RELB, TNFAIP3, EDA
LLC	Ptgs2, Lif, Junb, Fos, Traf5, Ccl20, Atf4, Creb5, Traf1, Mmp9, Irf1	Ptgs2, Fosb, Fos, Traf5, Ccl20, Fosl1, Mmp9	Nr4a1, Dusp2, Ereg, Dusp5, Srf, Fos, Areg, Atf4, Relb, Dusp1, Prkcg, Map3k12, Efna4, Efna1	Ptgs2, Plau, Traf5, Relb, Traf1, Syk, Btk

### 3.6 Tricin obviously downregulated the protein levels of SRC phosphorylation, JUNB, FOSB, and the immune checkpoint PD-L1, along with the classic proteins of the MAPK pathway, in PDGF-BB-stimulated KRAS^G12C^-mutant NSCLC cells

Western blotting was performed to determine the effects of tricin on downstream targets of SRC, including JUNB, FOSB, and DUSP2, and the immune checkpoint PD-L1 in KRAS^G12C^-mutant cell lines. It has been reported that the MAPK signaling pathway is the downstream of the SRC tyrosine kinase and it is the critical mediator of activator protein-1 (AP-1) transactivation (Whitmarsh and Davis, 1996; Li et al., 2018). The AP-1 complex consists of Jun family members (JUNB, c-JUN, JUND and v-JUN) and Fos family members (FOS, FOSB, FOSL1 and FOSL2), which directly promotes PD-L1 expression in tumor cells (Green et al., 2012). Therefore, the effects of tricin on the expression of classic proteins in the MAPK signaling pathway were also detected.

Considering the low levels of phosphorylated proteins in the SRC and MAPK pathways detected in KRAS^G12C^-mutant cells in the preliminary experiment, platelet-derived growth factor BB (PDGF-BB) was used for induction. First and foremost, Western blotting assay was used to detect the protein expressions of phosphorylation of SRC at Y416 and the phosphorylation of ERK1/2 at Thr 202/Tyr 204 in H358, H2122 and LLC cells, so as to validate the optimal concentration and time of PDGF-BB treatment. The results revealed that the optimal concentration and duration of PDGF-BB treatment for H358, H2122 and LLC cells were 100 ng/mL for 1.5 h, 75 ng/mL for 30 min and 100 ng/mL for 60 min, respectively (Supplementary Figure S3).

**FIGURE 5:**
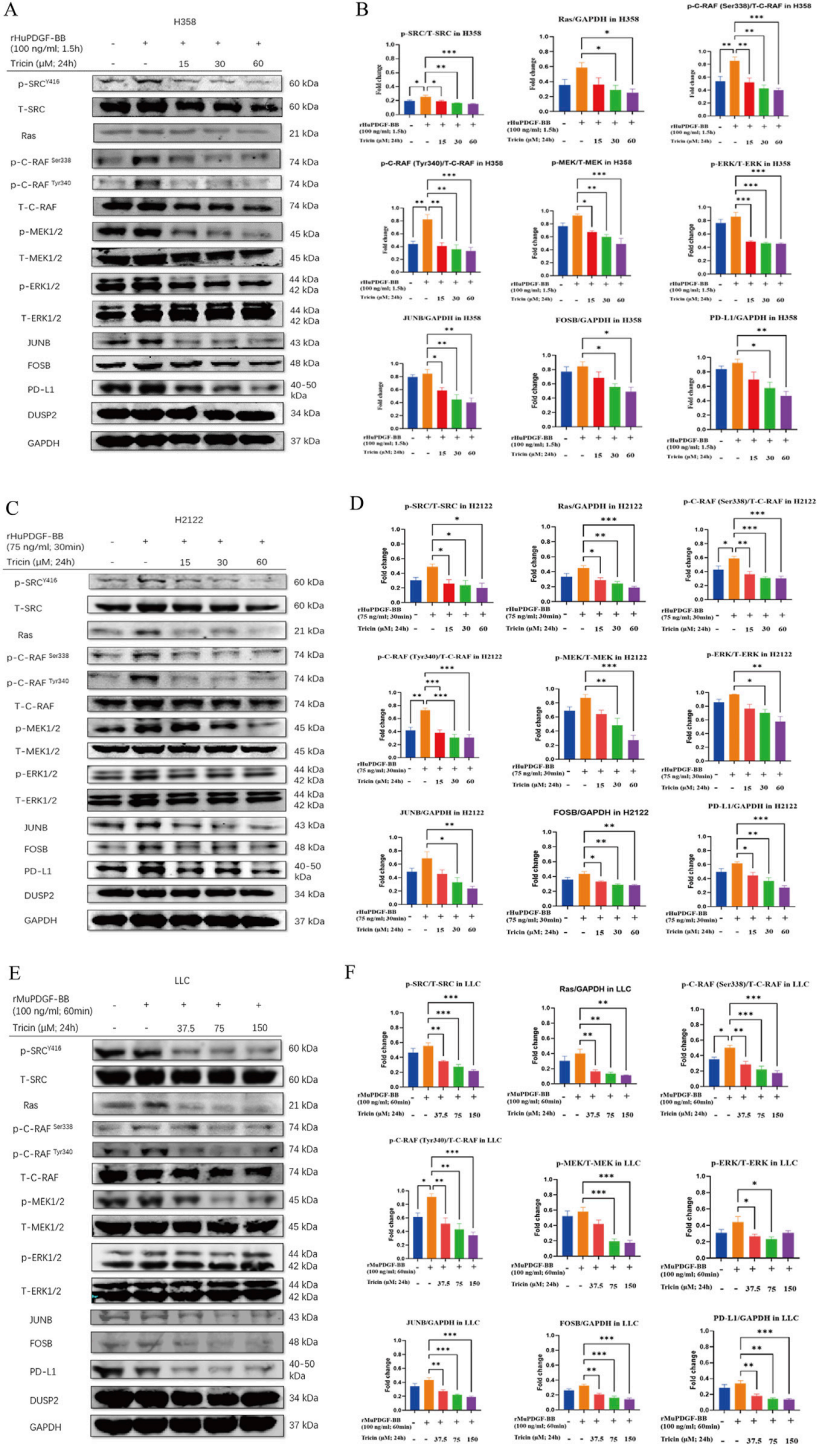
Tricin obviously downregulated the protein levels of SRC phosphorylation, JUNB, FOSB, and the immune checkpoint PD-L1, along with the classic proteins of the MAPK pathway, in PDGF-BB-stimulated KRAS^G12C^-mutant NSCLC cells. **(A,C,E)** After 24 h of tricin treatment along with different concentrations and time of PDFG-BB treatment, Western blotting assay was conducted to determine the protein expressions of p-SRC^Y416^, T-SRC, Ras, p-C-RAF^Ser338^, p-C-RAF^Tyr340^, T-C-RAF, p-MEK1/2, T-MEK1/2, p-ERK1/2, T-ERK1/2, JUNB, FOSB, PD-L1 and DUSP2 in H358, H2122 and LLC cells. **(B,D,F)** The statistical analysis of data about Western blot assay was conducted. Data were presented as mean ± SEM (*P < 0.05, **P < 0.01, ***P < 0.001vs. PDGF-BB induced cells).

Then, the protein expression levels of JUNB, FOSB, DUSP2, PD-L1 and classical proteins in the MAPK signaling pathway in H358, H2122 and LLC cells were subsequently monitored by Western blotting after tricin treatment and PDGF-BB induction (Figures 5A–F). In the rHuPDGF-BB-induced human KRAS^G12C^-mutant NSCLC cell lines H358 and H2122, the phosphorylation of SRC, C-RAF, MEK1/2, and ERK1/2 and the expressions of Ras, JUNB, FOSB and PD-L1 were clearly inhibited by increasing concentrations of tricin. The most effective concentration of tricin was 60 μM for 24 h. For the rMuPDGF-BB-induced murine KRAS^G12C^-mutant NSCLC cell line LLC, the phosphorylation of SRC, C-RAF, MEK1/2 and the expressions of Ras, JUNB, FOSB and PD-L1 were also obviously suppressed by increasing concentrations of tricin. However, the phosphorylation of ERK1/2 was not inhibited by the higher concentration of tricin (150 μM for 24 h). Thus, the most effective concentration of tricin was 75 μM for 24 h. In addition, DUSP2 protein expression was nearly unchanged after tricin treatment in all three cell lines. These results indicated that tricin distinctly inhibited the SRC phosphorylation and the protein expression of JUNB, FOSB, and the immune checkpoint PD-L1, along with the classic proteins in the MAPK pathway, in a dose-dependent manner in PDGF-BB-stimulated KRAS^G12C^-mutant NSCLC cells.

### 3.7 The inhibition of KRAS^G12C^-mutant NSCLC cell growth by tricin was associated with the crucial mediator SRC

**FIGURE 6:**
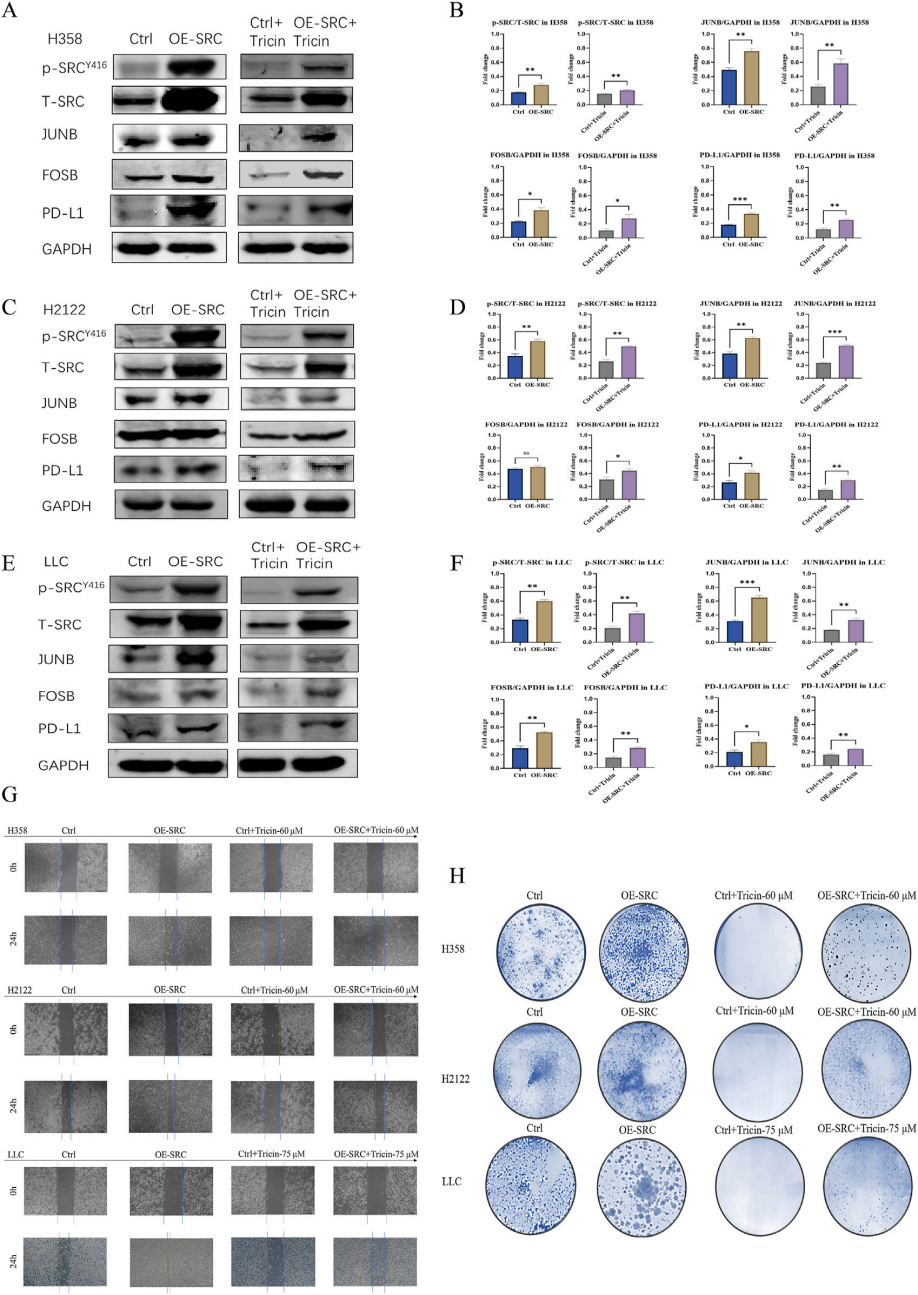
The inhibition of KRAS^G12C^-mutant NSCLC cell growth by tricin was associated with the crucial mediator SRC. **(A,C,E)** Western blotting assay was conducted to determine the protein expressions of SRC phosphorylation, JUNB, FOSB and PD-L1 in cells overexpressing SRC and control cells. Meanwhile, after 24 h of tricin treatment, Western blotting assay was also used to determine the protein expressions of SRC phosphorylation, JUNB, FOSB and PD-L1 in cells overexpressing SRC and control cells. **(B,D,F)** The statistical analysis of data about Western blot assay was conducted. Data were presented as mean ± SEM (*P < 0.05, **P < 0.01, ***P < 0.001). **(G)** The cell migration of cells overexpressing SRC and control cells treated with corresponding concentration of tricin at 24 h were exhibited. **(H)** The colony formation of cells overexpressing SRC and control cells treated with corresponding concentration of tricin were shown.

Moreover, we further verified whether tricin critically influenced the key target SRC during cancer cell growth and probed the underlying mechanism by overexpressing SRC in H358, H2122 and LLC cells via stable transfection (Supplementary Figures S4, S5). First, we examined the levels of phosphorylated SRC, JUNB, FOSB and PD-L1 in cells overexpressing SRC and control cells by immunoblotting. As shown in Figures 6A–F, the levels of SRC phosphorylation, JUNB, FOSB and PD-L1 all presented an increasing trend in KRAS^G12C^-mutant NSCLC cells overexpressing SRC. Moreover, the overexpression of SRC in H358, H2122 and LLC cells attenuated the downregulation of SRC phosphorylation and JUNB, FOSB and PD-L1 expression induced by tricin. In addition, as shown in Figures 6G,H, the overexpression of SRC obviously altered the inhibitory effects of tricin on cell migration and colony formation. Hence, SRC stood a good chance to be a crucial target of tricin-induced tumor cell suppression. The SRC/MAPK/AP-1/PD-L1 signaling pathway could be an important mechanism by which tricin confronted the KRAS^G12C^-mutant NSCLC.

### 3.8 Combined treatment with tricin and an anti-PD-1 antibody markedly inhibited the growth of tumors and altered the expression of key proteins in tumor tissues

**FIGURE 7:**
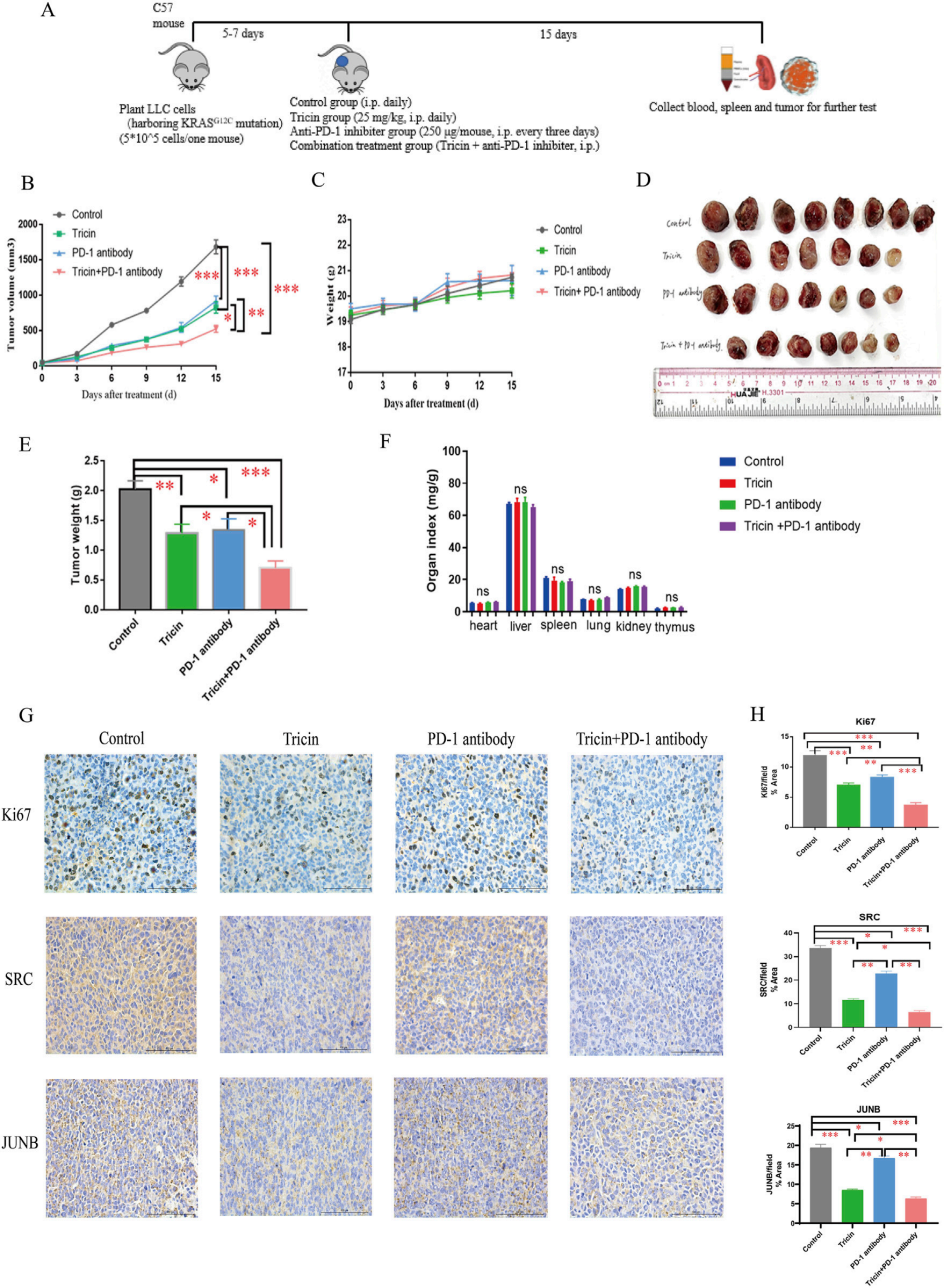
Combined treatment with tricin and an anti-PD-1 antibody markedly inhibited the growth of tumors and altered the expression of key proteins in tumor tissues. **(A)** The brief workflow of *in vivo* experiment was exhibited. **(B)** The line plot showed tumor volumes in each group of mice measured every 3 days. Comparisons were made among groups (*P < 0.05, **P < 0.01, ***P < 0.001). **(C)** The line plot displayed body weights of mice in each group measured every 3 days. **(D)** The photo about tumor tissues harvested from each group after 15 days of treatment was shown. The maximal tumor size was not exceeded ethics committee’s regulation. **(E)** Statistical bar graph of tumor weights in each group was exhibited. All data were presented as mean ± SEM (*P < 0.05, **P < 0.01, ***P < 0.001). **(F)** The bar graph about organ indexes of C57 mice treated with different drugs by intraperitoneal injection was shown. ns: not significant. **(G,H)** The immunochemistry staining about expressions of Ki67, SRC and JUNB were conducted. Images were taken with Nikon Eclipse E600 (×400 magnification, scale bar = 100 µm). Statistical analyses of data comparing tricin-treated tumor tissues, PD-1 antibody-treated tumor tissues, combined therapy-treated tumor tissues to normal saline-treated tissues were made. All data were presented as mean ± SEM (*P < 0.05, **P < 0.01, ***P < 0.001).

After the *in vitro* experiments were complete, *in vivo* experiments were also performed. In our previous study, both the lower-dose (25 mg/kg) and higher-dose of tricin (50 mg/kg) suppressed lung cancer growth without reducing the body weights of the mice (Li J.-X. et al., 2021). Given the immunotherapy resistance observed in patients with KRAS-mutated NSCLC and the ongoing clinical trials of KRAS^G12C^ inhibitors combined with immunotherapy, the pharmaceutical effect of a combination therapy with tricin and an anti-PD-1 inhibitor was explored in a mouse LLC cell xenograft model. When the tumors reached approximately 50–100 mm^3^ in size, the mice were randomized into four groups: the control group, the tricin group, the anti-PD-1 inhibitor group, and the combination group. Figure 7A exhibited the workflow of this experiment. As shown in Figure 7C, the body weights of the mice were not substantially affected by tricin, the PD-1 antibody and the combination therapy. Figures 7B,D showed that compared with tricin or the anti-PD-1 inhibitor alone, combined treatment with tricin and the PD-1 antibody markedly inhibited the growth of tumors. Specifically, the bar graph displayed that the tumor inhibition rates of tricin, the PD-1 antibody and the combination therapy were 39.32% (P < 0.01), 33.68% (P < 0.05), and 65.21% (P < 0.001), respectively (Figure 7E). Moreover, the toxic effects of tricin, the anti-PD-1 antibody and combination treatment on organs were tested. The results revealed no obvious effects of tricin, the PD-1 antibody or the combination treatment on the heart, liver, spleen, lung, kidney and thymus of the mice (Figure 7F). These results indicated that the combination therapy had no obvious toxicity to organs and had increased antitumor efficiency.

In addition, an immunohistochemical assay was performed to detect the expression of proteins associated with cell proliferation, differentiation and transcription in tumor tissues. Ki67 is a nuclear protein and a marker of cell proliferation in tumors. As shown by the images of Ki67 immunostaining in Figures 7G,H, tricin, the PD-1 antibody and the combination treatment decreased tumor cell proliferation. Compared with tricin or the anti-PD-1 inhibitor alone, combined treatment with tricin and the anti-PD-1 antibody markedly inhibited tumor cell proliferation. SRC and JUNB were core targets that have been verified *in vitro*. *In vivo*, the combined therapy markedly reduced the expressions of SRC and JUNB in tumor tissues. Tricin appeared to be more effective than the PD-1 antibody (Figures 7G,H). Thus, the effect of tricin *in vivo* was similar to that *in vitro*.

### 3.9 Tricin significantly increased the numbers of CD8^+^ T lymphocytes and B lymphocytes and regulated the PD-1/PD-L1 pathway, potentiating the antitumor effect of immunotherapy

**FIGURE 8:**
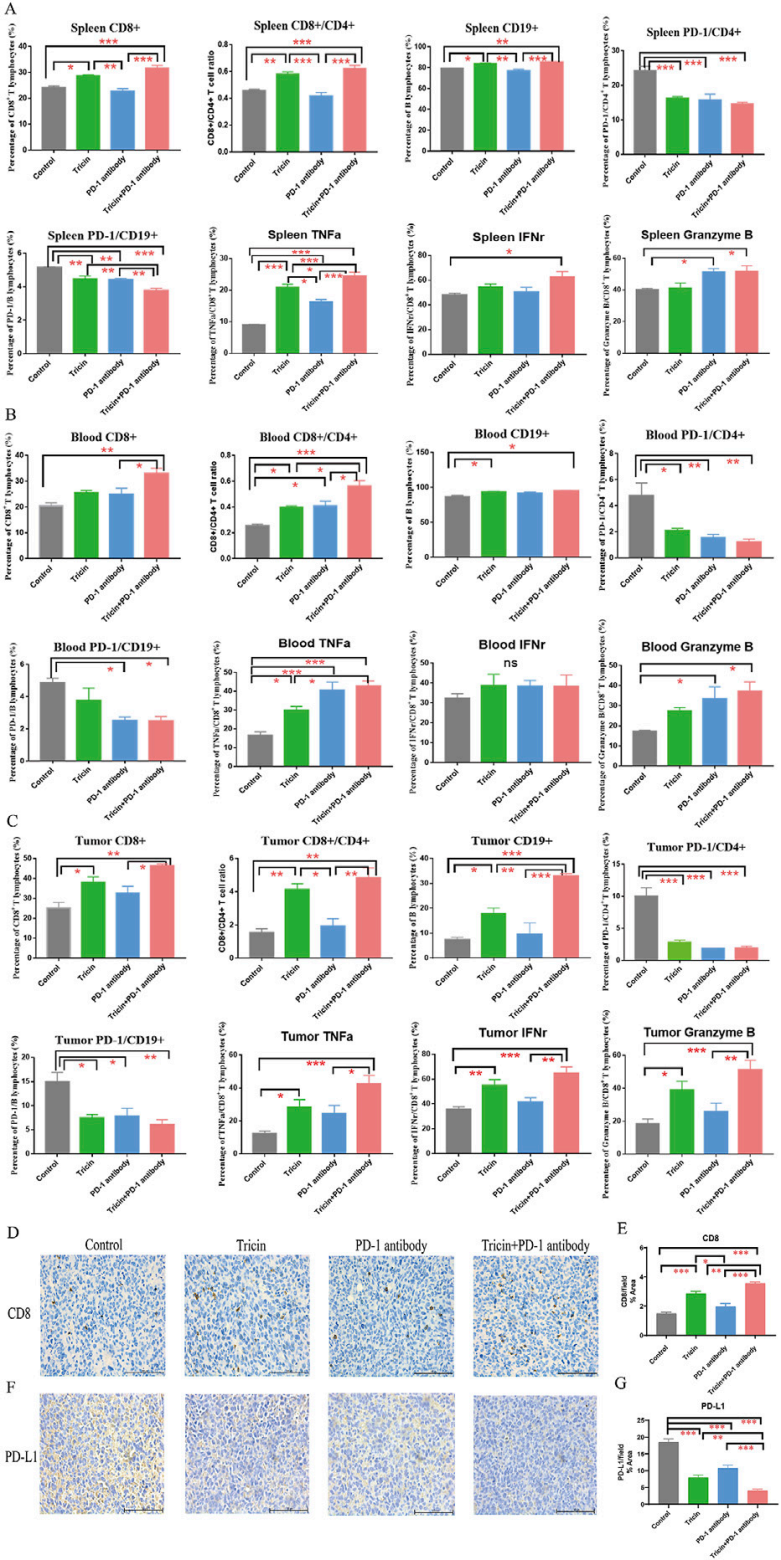
Tricin significantly increased the numbers of CD8^+^ T lymphocytes and B lymphocytes and regulated the PD-1/PD‐L1 pathway, potentiating the antitumor effect of immunotherapy. **(A–C)** Flow cytometry assay showed that the combinational treatment increased the percentages of CD8^+^ T cells and B lymphocytes, along with the ratio of CD8^+^/CD4^+^ T cell, decreasing the expressions of PD‐1 on CD4^+^ T cells and B lymphocytes in spleen, blood and tumor. Meanwhile, the combinational treatment also elevated the percentage of functional cytokines TNFα, IFNγ, and Granzyme B of CD8^+^ T cells in spleen, blood, and tumor. All data were presented as mean ±SEM (*P <0.05, **P <0.01, ***P <0.001). **(D–G)** The immunochemistry staining about expressions of CD8 and PD-L1 were conducted. Images were taken with Nikon Eclipse E600 (×400 magnification, scale bar=100 μm). Statistical analyses of data comparing tricin-treated tumor tissues, PD‐1 antibody-treated tumor tissues, combined therapy‐treated tumor tissues to normal saline‐treated tissues were made. All data were presented as mean ±SEM (*P <0.05, **P <0.01, ***P <0.001).

Flow cytometry and immunohistochemistry were used to verify whether tricin played a role in regulating immune function and its role in combination therapy. Supplementary Figure S6 exhibited representative charts of the flow cytometry assay. The flow cytometry data (Figures 8A–C) revealed that the percentages of CD8^+^ T cells and B lymphocytes, along with the ratio of CD8^+^/CD4^+^T cell in the spleen, blood and tumor were elevated by both tricin and the combination treatment. In contrast, the PD-1 antibody had little effect. The percentages of the functional cytokines TNFα, IFNγ, and Granzyme B in CD8^+^ T cells in the spleen, blood, and tumor were also obviously increased in response to the combination treatment. Moreover, the expressions of PD-1 on CD4^+^ T cells and B lymphocytes were significantly lower in the tricin group, PD-1 antibody group and combination treatment group than in the control group. Other parameters, such as the percentage of NK cells and the expression of PD-1 on CD8^+^ T cells and NK cells, were not obviously altered by these treatments.

The results of the immunohistochemical assay (Figures 8D–G) revealed that the combination therapy markedly increased the expression of CD8 and reduced the expression of PD-L1 in tumor tissues. Tricin seemed more effective than the PD-1 antibody. Therefore, these results indicated that tricin significantly increased the numbers of CD8^+^ T lymphocytes and B lymphocytes and regulated the PD-1/PD-L1 pathway, compensating for the deficiency of immunotherapy.

### 3.10 SRC expression had higher positive rate in elderly patients with early-stage KRAS-mutant NSCLC and was positively correlated with PD-L1 expression in tumors

Finally, the clinical significance of the related targets SRC, JUNB, and FOSB, along with PD-L1, in patient with KRAS-mutant NSCLC was investigated. The immunohistochemistry images revealed that SRC was located mainly in the cytoplasm of the tumor cells; JUNB and FOSB were located mainly in the cell nucleus; and PD-L1 was located mainly in the cytoplasm and nucleus of the tumor cells (Supplementary Figure S7). Table 2 showed that the positive rates of SRC, JUNB, FOSB and PD-L1 expression in patients with early-stage KRAS-mutant tumors were obviously higher than those in patients with advanced NSCLC (P < 0.05 and P < 0.01). Moreover, the rates of SRC positivity in elderly patients with KRAS-mutant NSCLC (>65 years old) were significantly greater than those in younger patients (P < 0.05). Meanwhile, the JUNB positive rate of male smoker with KRAS-mutant NSCLC was remarkably increased (P < 0.05). Additionally, as shown in table 3, SRC expression was positively correlated with PD-L1 expression in tumors (P = 0.001). Therefore, this clinical study provided a theoretical basis for clinicians to make decisions on precision medicine and formulate subsequent immune checkpoint combination therapy strategies.

**TABLE 2:** Association of the expressions of SRC, JUNB, FOSB and PD-L1 as well as clinicopathological characteristics in tumor samples of KRAS-mutant NSCLC patients.

Characteristics	SRC expression				JUNB expression				FOSB expression				PD-L1 expression			
	Positive (%)	Negative (%)	χ^2^	P value	Positive (%)	Negative (%)	χ^2^	P value	Positive (%)	Negative (%)	χ^2^	P value	Positive (%)	Negative (%	χ^2^	P value
Gender			0.000	1.000			5.714	**0.017**			1.000	0.317			0.229	0.633
Male	12 (66.7)	6 (33.3)			13 (72.2)	5 (27.8)			13 (72.2)	5 (27.8)			10 (55.6)	8 (44.4)		
Female	4 (66.7)	2 (33.3)			1 (16.7)	5 (83.3)			3 (50.0)	3 (50.0)			4 (66.7)	2 (33.3)		
Age (years)			4.000	**0.046**			0.229	0.633			0.067	0.795			0.229	0.633
≤65	2 (33.3)	4 (66.7)			3 (50.0)	3 (50.0)			4 (66.7)	2 (33.3)			3 (50.0)	3 (50.0)		
>65	14 (77.8)	4 (22.2)			11 (61.1)	7 (38.9)			13 (72.2)	5 (27.8)			11 (61.1)	7 (38.9)		
Smoking history			0.336	0.562			4.608	**0.032**			0.336	0.562			0.734	0.392
Smoker	8 (72.7)	3 (27.3)			9 (81.8)	2 (18.2)			8 (72.2)	3 (27.3)			7 (63.6)	4 (36.4)		
Nonsmoker	8 (61.5)	5 (38.5)			5 (38.5)	8 (61.5)			8 (61.5)	5 (38.5)			6 (46.2)	7 (53.8)		
Cell differentiation			0.086	0.770			0.490	0.484			0.086	0.770			3.311	0.069
Poorly	9 (64.3)	5 (35.7)			9 (64.3)	5 (35.7)			9 (64.3)	5 (35.7)			6 (42.9)	8 (57.1)		
Moderate and well	7 (70.0)	3 (30.0)			5 (50.0)	5 (50.0)			7 (70.0)	3 (30.0)			8 (80.0)	2 (20.0)		
TNM stage			4.200	**0.040**			5.531	**0.019**			4.200	**0.040**			7.073	**0.008**
I + II	9 (90.0)	1 (10.0)			9 (90.0)	1 (10.0)			9 (90.0)	1 (10.0)			9 (83.3)	1 (16.7)		
III + IV	7 (50.0)	7 (50.0)			6 (42.9)	8 (57.1)			7 (50.0)	7 (50.0)			5 (25.0)	9 (75.0)		

SRC, non-receptor tyrosine kinase; JUNB, AP-1 transcription factor subunit; FOSB, AP-1 transcription factor subunit; PD-L1, programmed cell death-ligand protein 1; TNM, tumor node metastasis. The bold values represent that p value is smaller than 0.05.

**TABLE 3:** Association of the expressions of SRC, JUNB and FOSB as well as PD-L1 expression in tumor samples of KRAS-mutant NSCLC patients.

PD-L1 expression	SRC expression				JUNB expression				FOSB expression			
	Positive	Negative	χ^2^	P value	Positive	Negative	χ^2^	P value	Positive	Negative	χ^2^	P value
Positive	13	1	10.371	**0.001**	10	4	2.371	0.124	10	4	0.343	0.558
Negative	3	7			4	6			6	4		

SRC, non-receptor tyrosine kinase; JUNB, AP-1 transcription factor subunit; FOSB, AP-1 transcription factor subunit; PD-L1, programmed cell death-ligand protein 1. The bold value represents that p value is smaller than 0.05.

## 4 Discussion and conclusions

**FIGURE 9:**
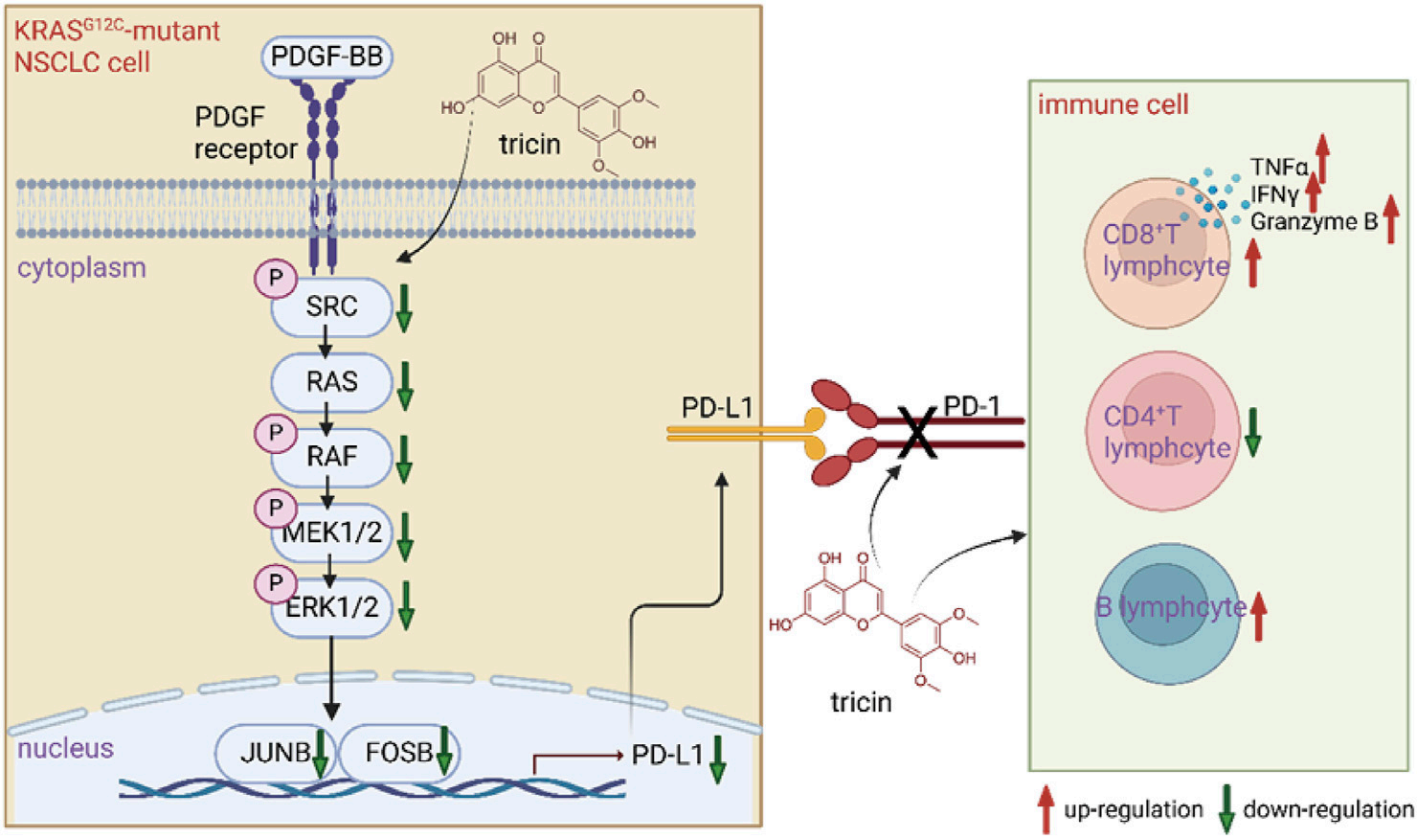
A diagram of the mechanisms by which tricin fighting against KRAS-mutant NSCLC. Tricin inhibited the growth of KRAS^G12C^-mutant NSCLC primarily by suppressing PDGF-BB-induced SRC/MAPK/AP-1/PD-L1 signaling pathway. On the other hand, tricin facilitated the increased number of CD8^+^ T lymphocytes along with functional cytokines TNFα, IFNγ, and Granzyme B. Tricin also increased the number of B lymphocytes as well as broke the PD-1/PD-L1 pathway.

Although several FDA-approved drugs are available for the treatment of KRAS-mutated NSCLC, these drugs are too expensive. Patients usually develop drug resistance and severe adverse effects. Fortunately, our previous study revealed that the natural compound tricin had selective cytotoxic effects on KRAS^G12C^-mutant tumor cell lines but had no significant effect on NSCLC cells with EGFR, ROS, or ALK mutations (Li J.-X. et al., 2021). This study is the first to integrate acute toxicity tests, bioinformatics analyses and transcriptomic analyses with various biological methods to understand the underlying mechanisms by which tricin combats KRAS-mutant NSCLC. Figure 9 exhibited a diagram of the mechanisms by which tricin against KRAS-mutant NSCLC growth vividly.

More specifically, we found that an intraperitoneal injection of tricin resulted in lower acute toxicity, as examined using the improved up-and-down procedure. The bioinformatics analysis revealed that tricin played important roles in inhibiting KRAS-mutated NSCLC growth by targeting hub genes such as SRC, PTGS2 and HIF1A. Moreover, tricin exhibited strong binding to SRC and PTGS2. Next, the results of functional experiments showed that tricin inhibited the migration, proliferation and colony formation of KRAS^G12C^-mutant NSCLC cells in a dose-dependent manner. Furthermore, according to the network pharmacology analysis, molecular docking and verification by real-time PCR, the SRC gene was selected as the main gene for our study. SRC is a proto-oncogene that participates in various processes, including the immune response, cell progression, cell apoptosis, cell migration, and gene transcription (GeneCards database). The abnormal activation of SRC is closely associated with the development of lung cancer, breast cancer, colon cancer, pancreatic cancer and other tumors (Summy and Gallick, 2003). The upregulation of receptor tyrosine kinases (RTKs) can induce abnormal activation of SRC, which then activates many signaling cascades related to tumor development, leading to tumor cell growth, transformation, migration and invasion. These various signaling cascades cover the Ras-MAPK, PI3K-AKT, JAK-STAT3 as well as FAK/Paxillin pathways (Raji et al., 2023). Various SRC inhibitors have been developed, and several of them have shown promise in completed as well as ongoing clinical trials. Dasatinib, Saracatinib and Bosutinib are considered to be the three most studied SRC inhibitors. Meanwhile, KX2–391, XL228, DCC-2036, AP24534 along with TG100435 are also inhibitors in development (Puls et al., 2011). By means of analyzing the data collected from TCGA, we indicated that our findings might have clinical implications. Moreover, transcriptome sequencing revealed that the common downstream targets of SRC in KRAS^G12C^-mutant NSCLC cells were JUNB, FOSB and DUSP2, which were related to inflammatory and immune pathways. Many studies have reported that the MAPK signaling pathway is downstream of SRC and is an important mediator of AP-1 transactivation (Whitmarsh and Davis, 1996; Li et al., 2018). The AP-1 complex comprises JUNB and FOSB, which directly induces the expression of PD-L1 in cancer cells (Green et al., 2012). With respect to these findings, we performed a Western blot assay. The results showed that tricin dose-dependently inhibited KRAS^G12C^-mutant NSCLC cell growth primarily by suppressing the PDGF-BB-induced SRC/MAPK/AP-1/PD-L1 signaling pathway. Moreover, assays using stably transfected cells further verified that SRC was likely a key target for tricin-induced inhibition of tumor cell growth.

After the *in vitro* experiments were complete, we also performed *in vivo* experiments. In our previous research, tricin was verified to suppress lung cancer growth in an LLC xenograft mouse model (Li J.-X. et al., 2021). It has been reported that patients with KRAS-mutant NSCLC can obtain the benefit from immunotherapy (Liu et al., 2020). However, they are usually resistant to anti-PD-1/PD-L1 therapy. The mechanisms of immunotherapy resistance are often due to low levels of tumor-infiltrating lymphocytes, including CD8^+^ T lymphocytes and B lymphocytes (Shergold et al., 2019; Bejarano et al., 2021). At the same time, several studies have found that immunosuppressive agents combined with other therapies, including some natural extracts, were often more effective than monotherapy (Huang et al., 2022b; Zhang et al., 2024). In the analysis of the efficacy of the combination therapy, we were pleased to find that it had no obvious toxicity to organs and had greater antitumor efficiency than tricin or the PD-1 antibody alone. With respect to the regulation of immunity, tricin increased the number of CD8^+^ T lymphocytes and the levels of the functional cytokines TNFα, IFNγ, and Granzyme B. Tricin also increased the number of B lymphocytes and disrupted the PD-1/PD-L1 pathway. Tricin could compensate for the deficiency of immunotherapy and enhance the antitumor activity of immunotherapy, thus facilitating the role of the combination treatment. Moreover, using IHC staining, we found that the effect of tricin *in vivo* was similar to that *in vitro*.

In addition to cell-based and animal experiments, clinical samples collected at Jiangsu Province Hospital of Chinese Medicine were also analyzed in our study. We found that the rate of SRC positivity was higher in elderly patients with early-stage KRAS-mutant NSCLC. Moreover, a positive correlation between the expression of SRC and PD-L1 in tumor tissues was observed. This study provides a theoretical basis for the future clinical translation of tricin and the development of subsequent immune checkpoint combination therapeutic strategies.

Like most studies, our study has several limitations and directions for further exploration. First, the natural flavonoid tricin has an oral bioavailability of only 27% and poor solubility, which may lead to a slow onset of action and limited efficacy in lung cancer patients. The self-nanoemulsifying drug delivery system (SNEDDS) is an important strategy for the delivery of drugs with low bioavailability and poor solubility in water and has the potential to improve transcellular permeability and lymphatic transport (Kim et al., 2014; Baheti et al., 2016; Park, 2017). It forms an oil/water emulsion suitable for drug delivery under mild agitation in GI fluid (Baheti et al., 2016). Moreover, SNEDDSs have the advantages of inhibiting efflux pumps, reducing first-pass metabolism, and quickly diffusing via the nanoemulsion (Friedl et al., 2013; Rohrer et al., 2016; Suchaoin et al., 2016; Zupančič et al., 2016). For instance, Professor Xie’s group has used total glucosides of peony and nobiletin coloaded within SNEDDSs to treat refractory rheumatoid arthritis (Qu et al., 2022). Thus, we can use tricin-loaded SNEDDSs for increased bioavailability in future studies. Second, a mutated KRAS protein has been reported to recruit proinflammatory macrophages to promote tumor growth in pancreatic cancer (Döppler and Storz, 2024). Our future research direction will also focus on exploring whether tricin can target other immune cells, such as macrophages, to inhibit KRAS-mutant NSCLC growth. Third, tumor infiltrating lymphocytes have been rapidly expanded to contain B cells and plasma cells recently, collectively known as TIL-Bs (Laumont and Nelson, 2023). Several studies have shown a strongly positive correlation between the presence of TIL-Bs and a good prognosis in many cancers as well as clinical response to immune checkpoint blockade therapy (Cabrita et al., 2020; Helmink et al., 2020; Petitprez et al., 2020; Vanhersecke et al., 2021). The specific role of tricin in TIL-Bs will be explored in depth in further studies. Moreover, a team of researchers from Rockefeller University used functional genomic and lipidomic methods to reveal the key role of sphingolipid synthesis in cancer immune escape. They reported that blocking sphingolipid production in KRAS-mutant pancreatic cancer cells, especially through the regulation of the IFNγ signaling pathway, could effectively increase the efficacy of natural killer cells and CD8^+^ T cells (Soula et al., 2024). These results suggest that we can use a variety of novel omics techniques, such as metabolomics and spatial transcriptomics, to explore the mechanism by which tricin potentiates the antitumor effect of immune checkpoint inhibitors on KRAS-mutant NSCLC.

Taken together, we believe that tricin is a developable agent for the treatment of patients with KRAS-mutant NSCLC.

## Data Availability

The original contributions presented in the study are publicly available. Raw data have been deposited to National Center for Biotechnology Information (NCBI) under the BioProject number PRJNA1274524.
