# Supplementary material for: Tricin selectively combats KRAS-mutant non-small cell lung cancer by inhibiting the PDGF-BB-induced SRC/MAPK/AP-1/PD-L1 signaling pathway and potentiating the antitumor effect of an anti-PD-1 antibody
Source: Front Pharmacol. 2025 Jun 17;16:1594213. doi: 10.3389/fphar.2025.1594213 (PMC12209307; doi:10.3389/fphar.2025.1594213)
Supplement: Supplementary file 1 [file DataSheet1.pdf]

Supplementary figure and table

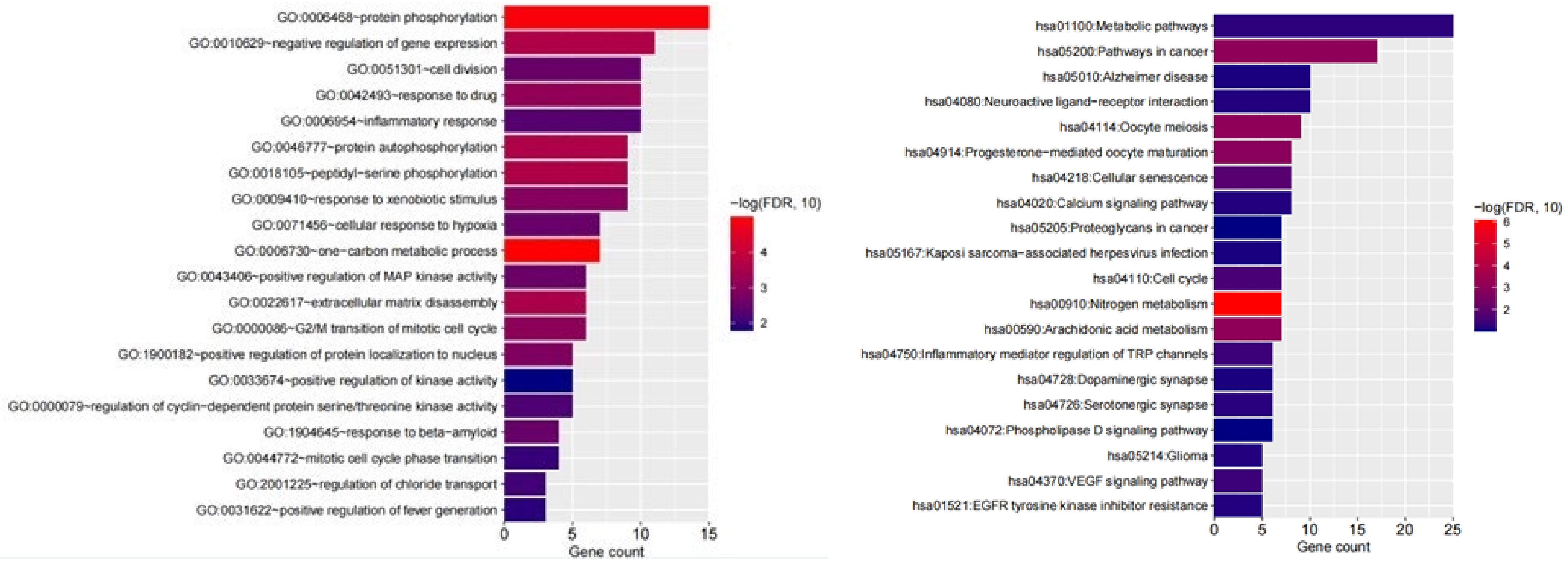

Supplementary figure 1. Main biological process and KEGG enrichment pathways of intersection protein targets. The vertical axis represented the function/pathway names, and the horizontal axis represented the number of enriched genes. The redder the color was, the more significant the enrichment was.

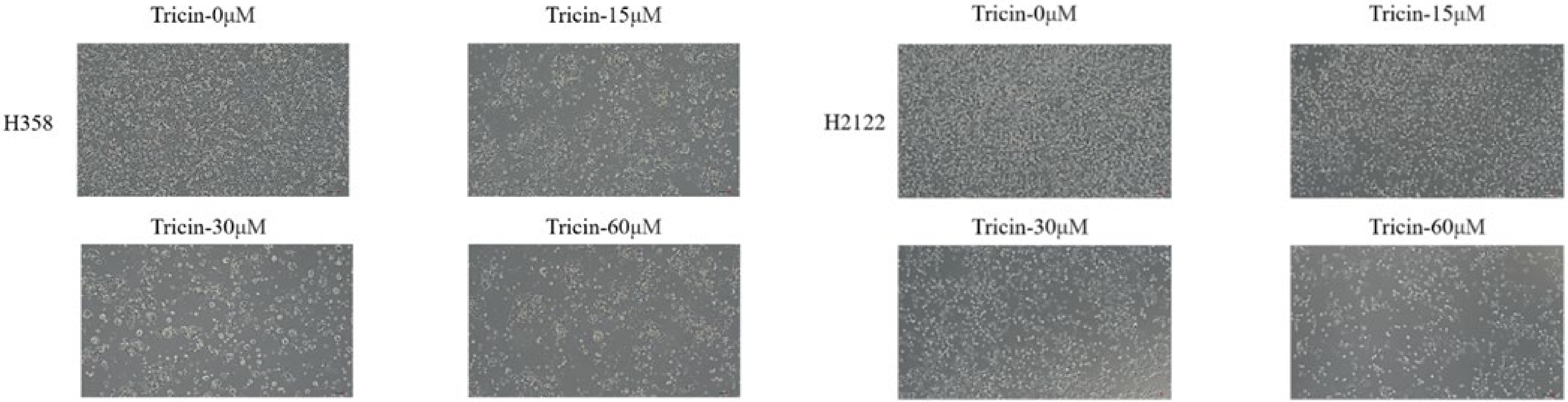

Supplementary figure 2. The viability of H358 and H2122 cells treated with different concentrations of tricin for 24h

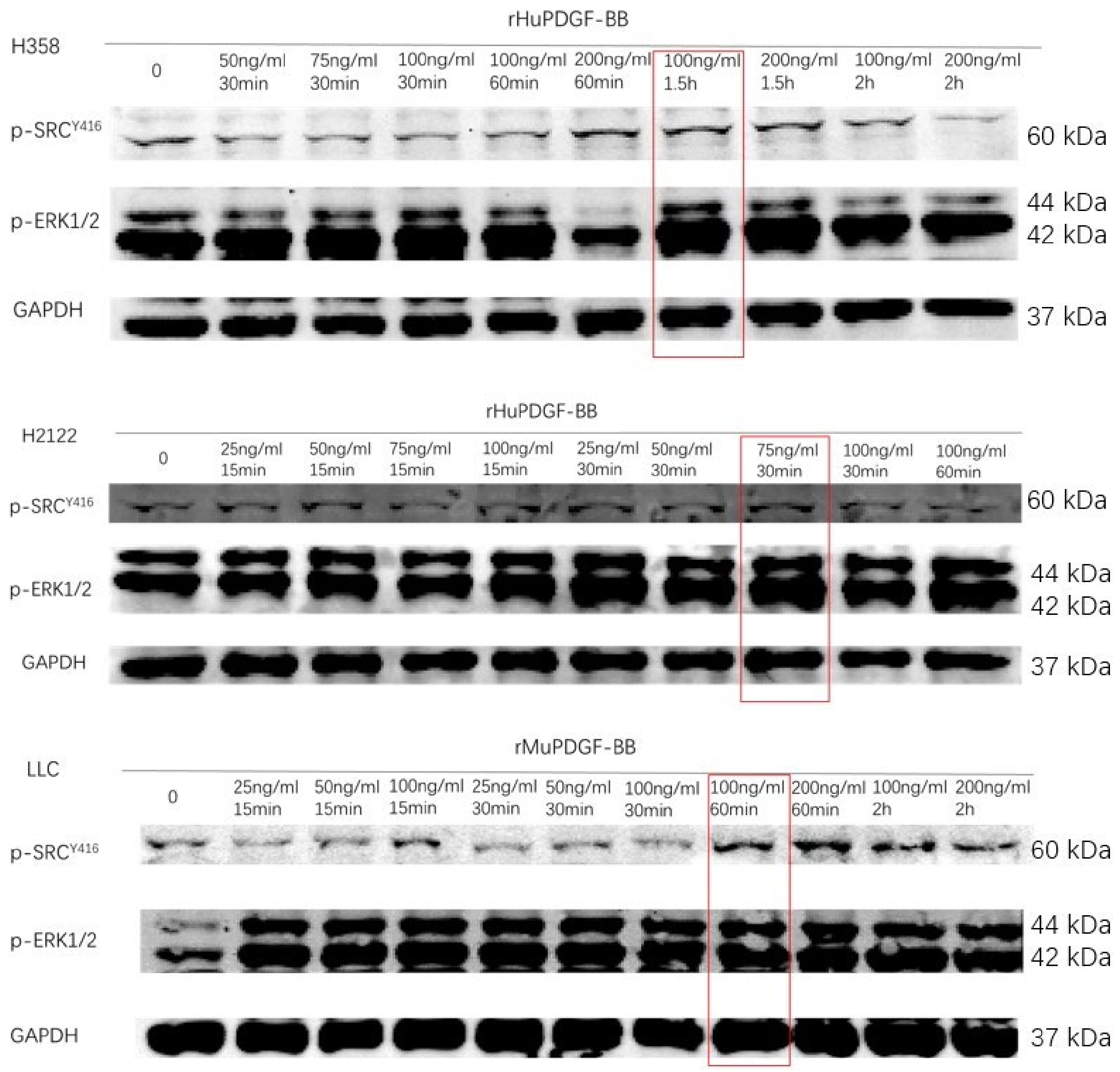

Supplementary figure 3. The protein effect of different concentrations and treatment time of PDGF-BB acted on H358, H2122 and LLC cells

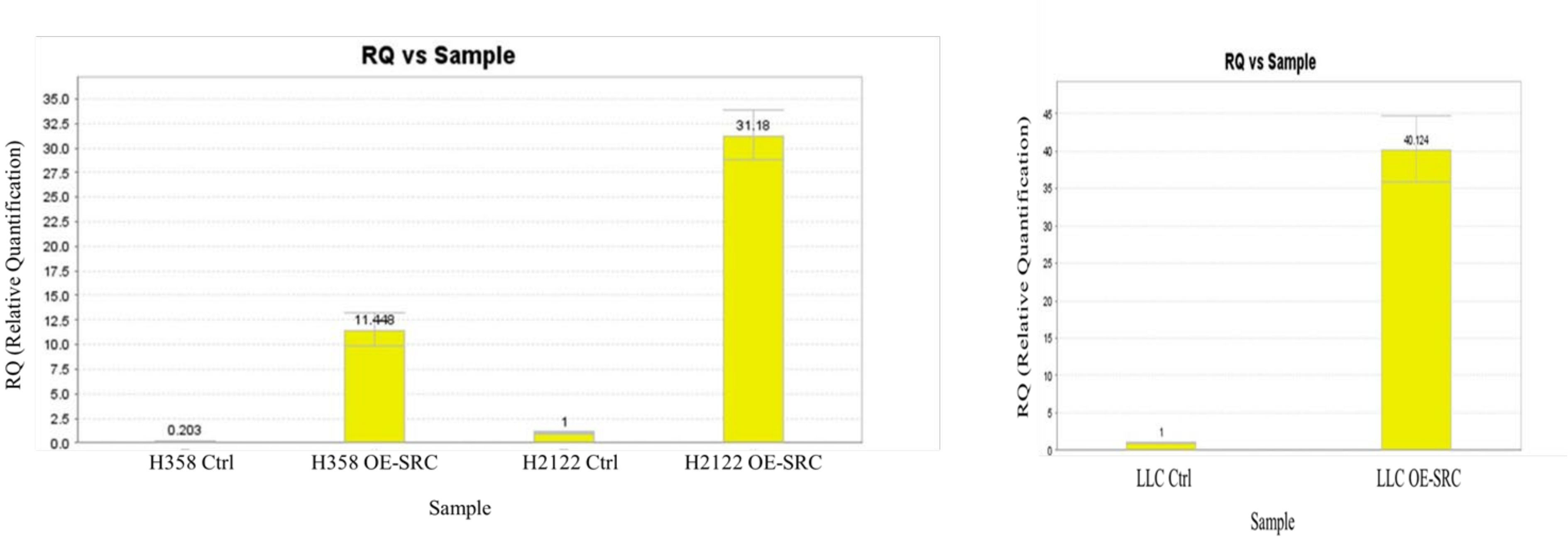

Supplementary figure 4. Quantitative PCR results showed that the mRNA expression levels of SRC gene were all significantly up-regulated following stable transfection

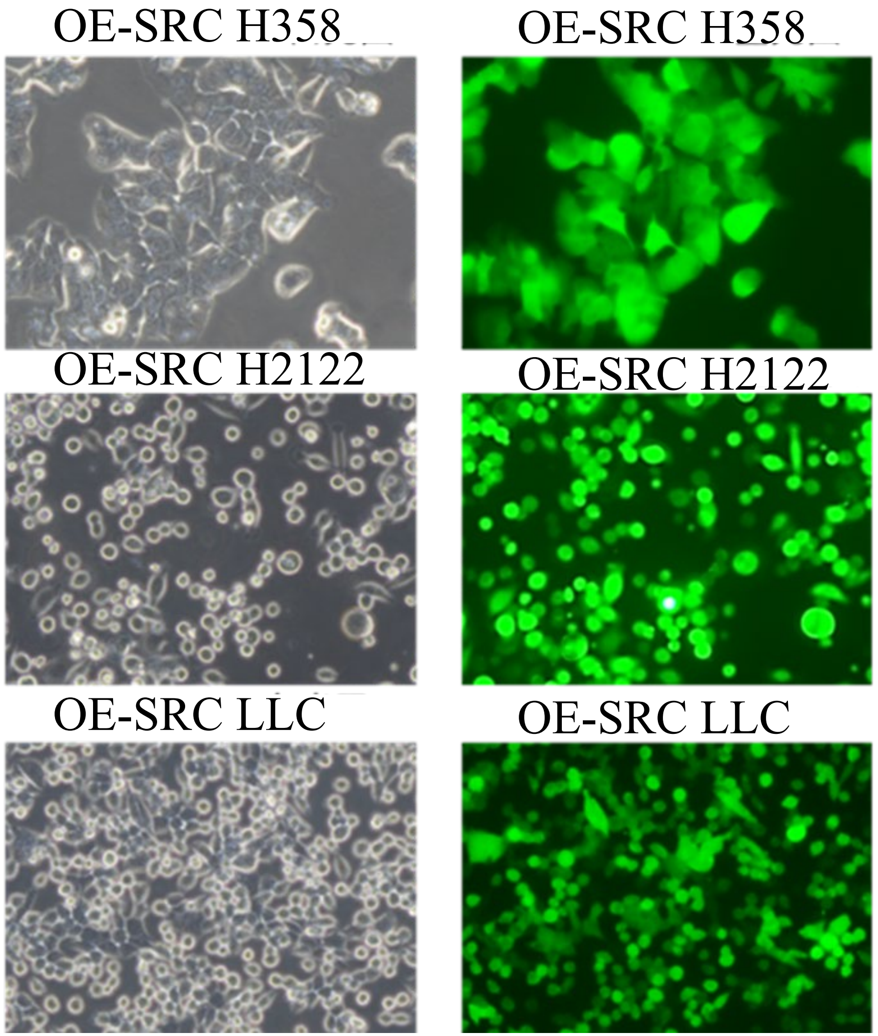

Supplementary figure 5. White light and fluorescence images of the three SRC-overexpressed cells were shown under fluorescence microscopy

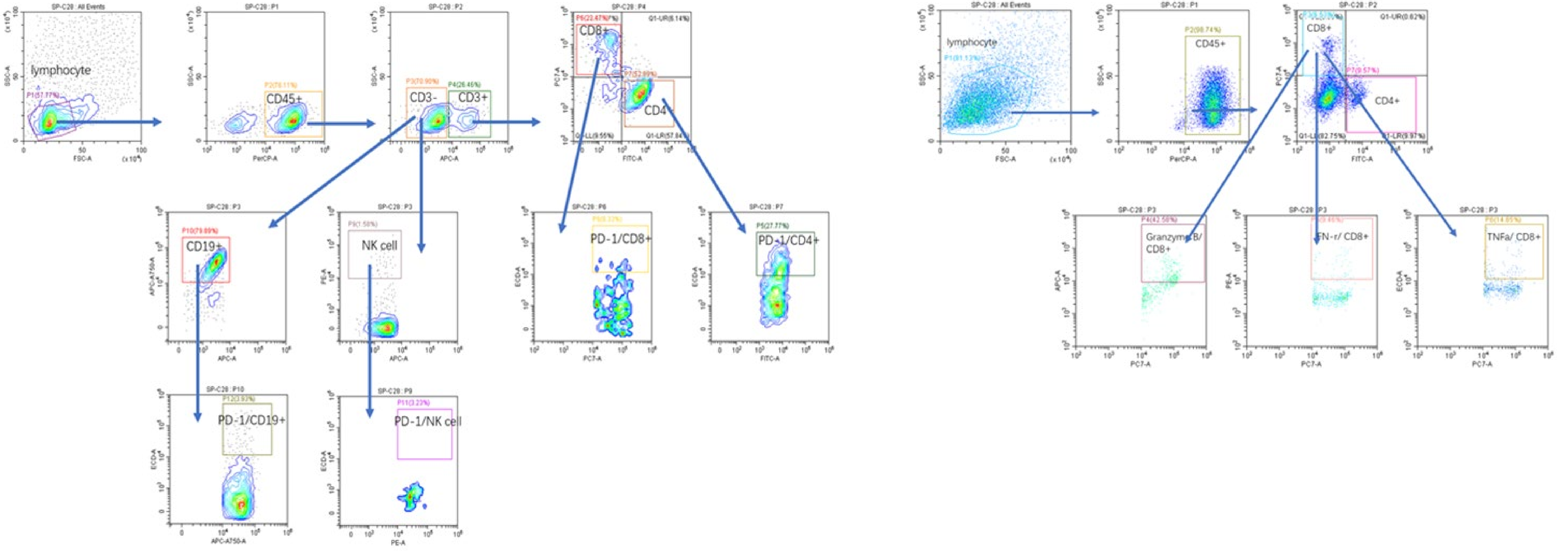

Supplementary figure 6. Representative flow charts of flow cytometry assay

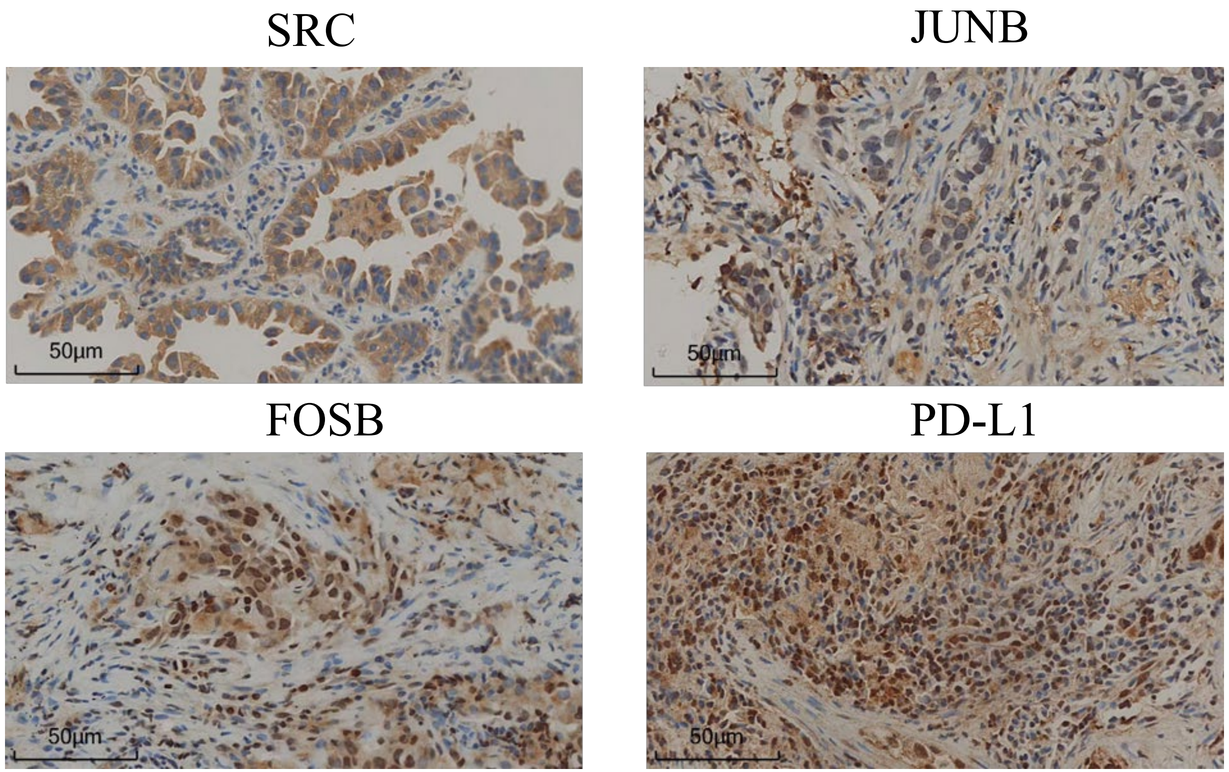

Supplementary figure 7. Images about the positive expressions of SRC, JUNB, FOSB and PD-L1 in tumor issues of KRAS-mutant NSCLC patients. Images were taken with TEKSQRAY (scale bar=50 μm).

Supplementary table 1. Main reagents relevant to the experiments

Name	Manufacturer
Tricin	MedChemExpress (New Jersey, USA)
Saracatinib	MedChemExpress (New Jersey, USA)
PDGF-BB protein, human	MedChemExpress (New Jersey, USA)
PDGF-BB protein, mouse	MedChemExpress (New Jersey, USA)
Pierce™ BCA Protein Assay Kit	Thermo Scientific™ (Waltham, Massachusetts, USA)
Fluorescein-conjugated rabbit antibody	Odyssey (Belfast, ME, USA)
MTT powder	Sigma Aldrich (St. Louis, MI, USA)
Phospho-Src Family (Tyr416) (D49G4) Rabbit mAb	Cell Signaling Technology (Danvers, MA, USA)
Src Rabbit mAb	Signalway Antibody (Maryland, USA)
Ras (27H5) Rabbit mAb	Cell Signaling Technology (Danvers, MA, USA)
Phospho-c-Raf (Ser338) (56A6) Rabbit mAb	Cell Signaling Technology (Danvers, MA, USA)
C-RAF (Phospho-Tyr340) Antibody	Signalway Antibody (Maryland, USA)
C-Raf Antibody	Cell Signaling Technology (Danvers, MA, USA)
MEK1/2 Antibody	Cell Signaling Technology (Danvers, MA, USA)
Phospho-MEK1/2 (Ser217/221) Antibody	Cell Signaling Technology (Danvers, MA, USA)
p44/42 MAPK (Erk1/2) Antibody	Cell Signaling Technology (Danvers, MA, USA)
Phospho-p44/42 MAPK (Erk1/2) (Thr202/Tyr204) Antibody	Cell Signaling Technology (Danvers, MA, USA)
JUNB Antibody	Signalway Antibody (Maryland, USA)
FosB Rabbit mAb	Signalway Antibody (Maryland, USA)
PD-L1/CD274 Antibody	Signalway Antibody (Maryland, USA)
DUSP2 Antibody	Signalway Antibody (Maryland, USA)
GAPDH Antibody	Signalway Antibody (Maryland, USA)
ReverTra Ace qPCR RT Master Mix with gDNA Remover	TOYOBO (Osaka, Japan)
FastStart Universal SYBR Green Master	Roche (Mannheim, Germany)
Anti-Mouse CD279 (PD-1) (Clone RMP1-14)–Purified in vivo GOLD™ Functional Grade	Leinco Technologies (St. Louis, Missouri, USA)
Immunohistochemistry kit	Servicebio (Wuhan, Hubei, China)
Ki-67 Rabbit mAb	Servicebio (Wuhan, Hubei, China)

Name	Manufacturer
CD8 Rabbit mAb	Servicebio (Wuhan, Hubei, China)
Invitrogen™ UltraComp eBeads™ Compensation Beads	Thermo Scientific™ (Waltham, Massachusetts, USA)
Foxp3/Transcription Factor Fixation/Permeabilization Concentrate and Diluent	Thermo Scientific™ (Waltham, Massachusetts, USA)
Permeabilization Buffer (10X)	Thermo Scientific™ (Waltham, Massachusetts, USA)
Red blood cell lysis buffer	Sigma Aldrich (St. Louis, MI, USA)
Cell Activation Cocktail (with Brefeldin A)	Biolegend (San Diego, CA, USA)
PE anti-mouse IFNγ	Biolegend (San Diego, CA, USA)
PE/Dazzle™ 594 anti-mouse TNFα	Biolegend (San Diego, CA, USA)
APC anti-human/mouse Granzyme B Recombinant	Biolegend (San Diego, CA, USA)
FITC anti-mouse CD4	Biolegend (San Diego, CA, USA)
PE/Cy7 anti-mouse CD8a	Biolegend (San Diego, CA, USA)
PerCP anti-mouse CD45	Biolegend (San Diego, CA, USA)
APC anti-mouse CD3	Biolegend (San Diego, CA, USA)
PE anti-mouse NK-1.1	Biolegend (San Diego, CA, USA)
APC/Cyanine7 anti-mouse CD19	Biolegend (San Diego, CA, USA)
PE/Dazzle™ 594 anti-mouse CD279 (PD-1)	Biolegend (San Diego, CA, USA)
Plasmid small extraction kit	GENERAY Biotechnology

Supplementary table 2. Sequences of PCR primers

Gene name	Race	Primer name	Sequence (5' to 3')
SRC	Human	hSRC-F	CTGCTTTGGCGAGGTGTGGATG
		hSRC-R	CCACAGCATACAACTGCACCAG
Src	Mouse	mSrc-F	GTTGCTTCGGAGAGGTGTGGAT
		mSrc-R	CACCAGTTTCTCGTGCCTCAGT
PTGS2	Human	hPTGS2-F	CGGTGAAACTCTGGCTAGACAG
		hPTGS2-R	GCAAACCGTAGATGCTCAGGGA
Ptgs2	Mouse	mPtgs2-F	GCGACATACTCAAGCAGGAGCA
		mPtgs2-R	AGTGGTAACCGCTCAGGTGTTG
HIF1A	Human	hHIF1A-F	TATGAGCCAGAAGAACTTTTAGGC
		hHIF1A-R	CACCTCTTTTGGCAAGCATCCTG
Hif1a	Mouse	mHif1a-F	CCTGCACTGAATCAAGAGGTTGC
		mHif1a-R	CCATCAGAAGGACTTGCTGGCT
GAPDH	Human	hGAPDH-F	GTCTCCTCTGACTTCAACAGCG
		hGAPDH-R	ACCACCCTGTTGCTGTAGCCAA
Gpadh	Mouse	mGpadh-F	CATCACTGCCACCCAGAAGACTG
		mGpadh-R	ATGCCAGTGAGCTTCCCGTTCAG

Supplementary table 3. Cancer-related pathways and hub targets

Pathway	Hub target
Pathways in cancer	CAMK2B, RET, NOS2, PRKCB, DAPK1, FLT3, PTGER3, PIK3R1, F2, PTGS2, HIF1A, MMP9, CCNA2, AR, CDK6, TERT, CALM1
Cellular senescence	CCNB3, CCNA2, CCNB2, CCNB1, CDK6, CDK1, PIK3R1, CALM1
Cell cycle	CCNB3, CCNA2, CCNB2, CCNB1, CDK6, PLK1, CDK1
Inflammatory mediator regulation of TRP channels	CAMK2B, SRC, PRKCB, ALOX12, PIK3R1, CALM1
VEGF signaling pathway	SRC, PRKCB, KDR, PIK3R1, PTGS2
Neuroactive ligand-receptor interaction	OPRD1, PRSS1, CNR2, ADORA2A, CNR1, PTGER3, AVPR2, PLG, F2, DRD4
EGFR tyrosine kinase inhibitor resistance	SRC, PRKCB, AXL, KDR, PIK3R1
Calcium signaling pathway	CAMK2B, RET, ADORA2A, NOS2, PRKCB, PTGER3, KDR, CALM1
Proteoglycans in cancer	CAMK2B, SRC, PRKCB, KDR, PIK3R1, HIF1A, MMP9
Phospholipase D signaling pathway	CXCR1, DGKA, AVPR2, PIK3R1, F2, PIK3CG
Rap1 signaling pathway	ADORA2A, CNR1, SRC, PRKCB, KDR, PIK3R1, CALM1
Oxytocin signaling pathway	CAMK2B, SRC, PRKCB, CALM1, PTGS2, PIK3CG
HIF-1 signaling pathway	CAMK2B, NOS2, PRKCB, PIK3R1, HIF1A
Ras signaling pathway	FLT3, PLA2G1B, PRKCB, PLA2G2A, KDR, PIK3R1, CALM1
Folate biosynthesis	CBR1, AKR1B10, AKR1B1
Pathways of neurodegeneration - multiple diseases	CAMK2B, CSNK2A1, NOS2, CDK5, PRKCB, NOX4, MAPT, CALM1, PTGS2, CDK5R1
Non-small cell lung cancer	RET, CDK6, PRKCB, PIK3R1
p53 signaling pathway	CCNB2, CCNB1, CDK6, CDK1
FoxO signaling pathway	CCNB3, CCNB2, CCNB1, PLK1, PIK3R1
Estrogen signaling pathway	SRC, PGR, PIK3R1, CALM1, MMP9
ErbB signaling pathway	CAMK2B, SRC, PRKCB, PIK3R1
Chemical carcinogenesis - reactive oxygen species	CBR1, SRC, NOX4, AKR1C4, PIK3R1, HIF1A

Uncropped raw image

H358

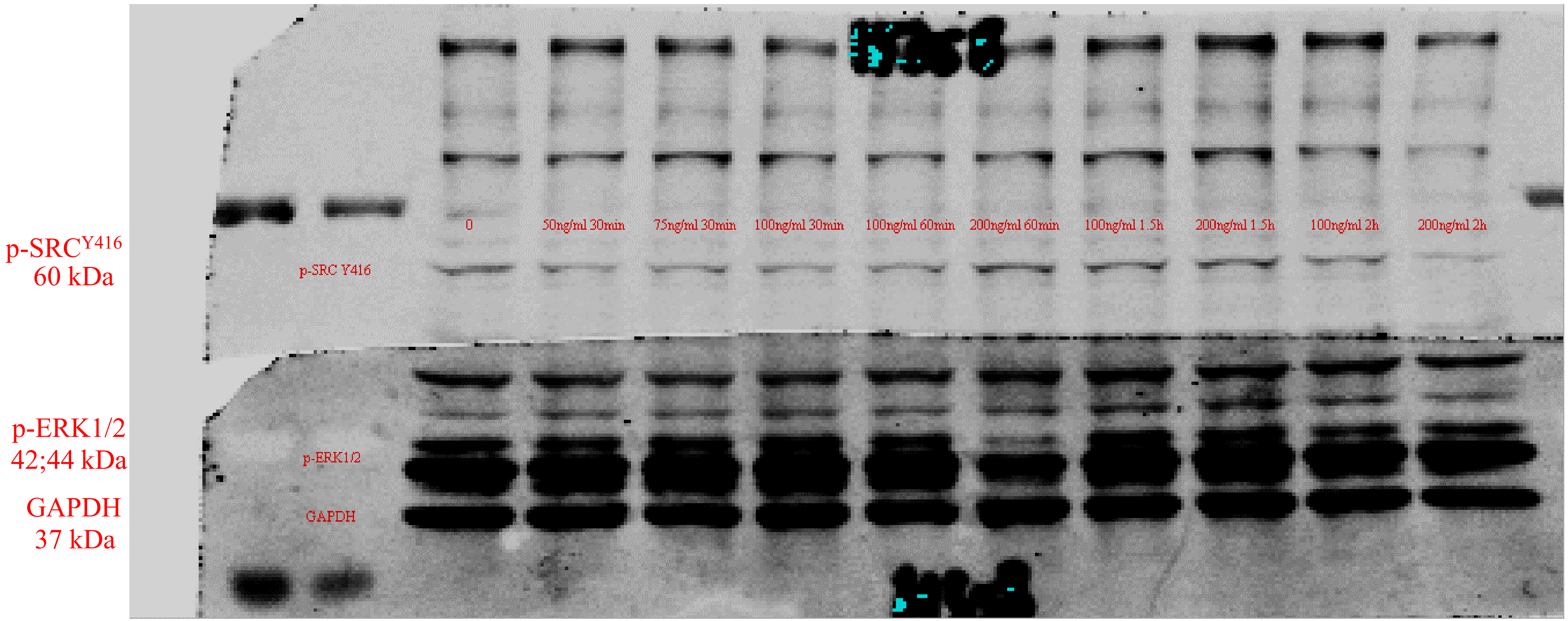
p-SRC^{Y416}
60 kDa
p-ERK1/2
42;44 kDa
GAPDH
37 kDa
0
50ng/ml 30min
75ng/ml 30min
100ng/ml 30min
100ng/ml 60min
200ng/ml 60min
100ng/ml 1.5h
200ng/ml 1.5h
100ng/ml 2h
200ng/ml 2h
p-SRC Y416
p-ERK1/2
GAPDH

H2122

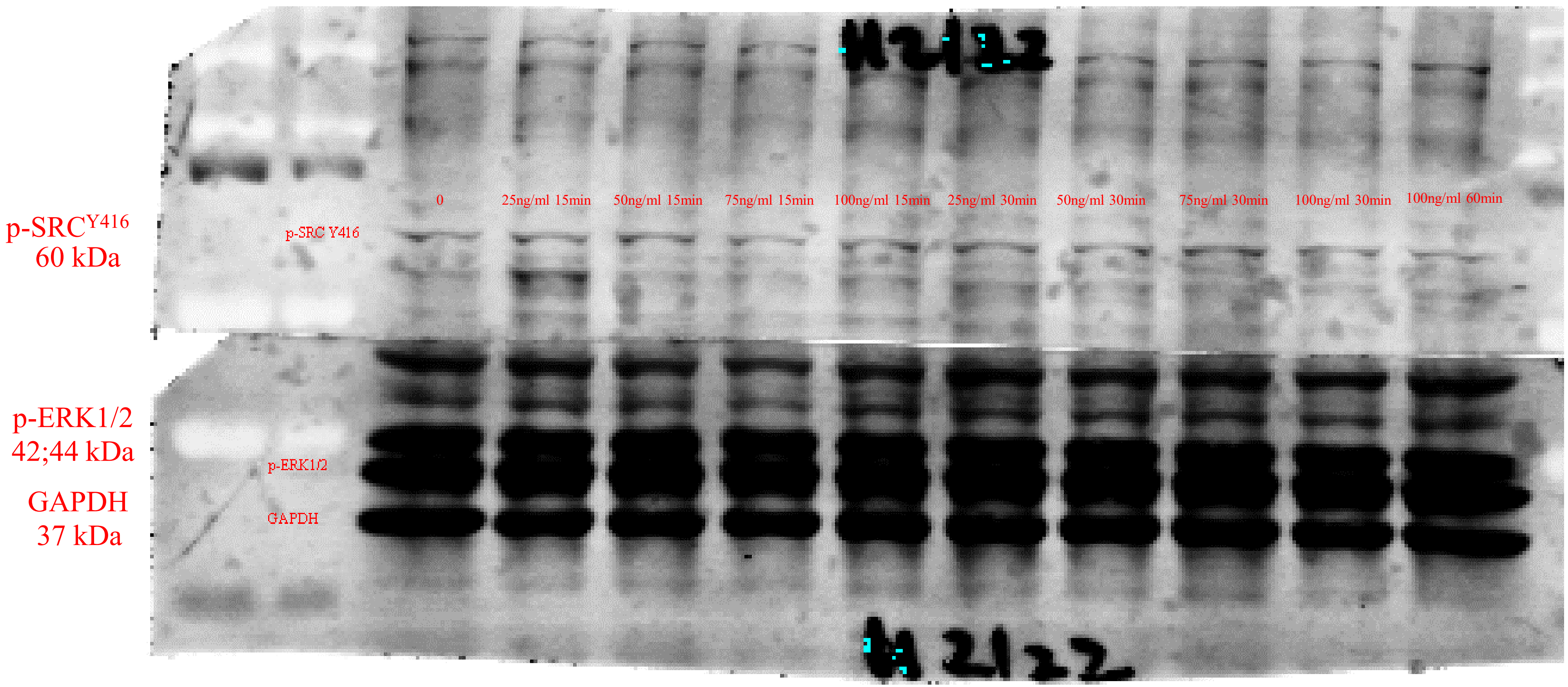

p-SRC Y416
60 kDa
p-ERK1/2
42;44 kDa
GAPDH
37 kDa
H2122
0
25ng/ml 15min
50ng/ml 15min
75ng/ml 15min
100ng/ml 15min
25ng/ml 30min
50ng/ml 30min
75ng/ml 30min
100ng/ml 30min
100ng/ml 60min
p-SRC Y416
p-ERK1/2
GAPDH
H2122

LLC

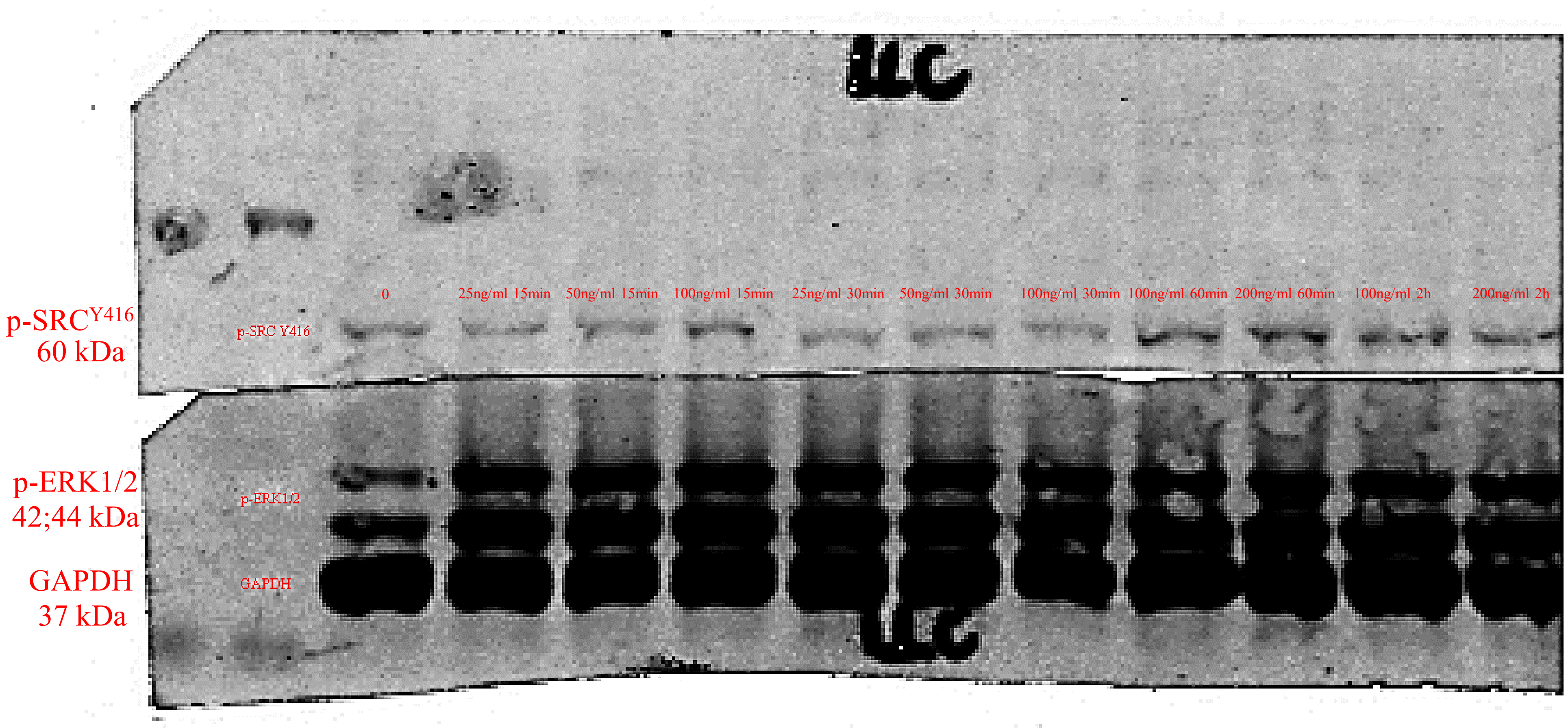
0
25ng/ml 15min
50ng/ml 15min
100ng/ml 15min
25ng/ml 30min
50ng/ml 30min
100ng/ml 30min
100ng/ml 60min
200ng/ml 60min
100ng/ml 2h
200ng/ml 2h
p-SRC^{Y416}
60 kDa
p-SRC Y416
p-ERK1/2
42;44 kDa
p-ERK1/2
GAPDH
37 kDa
GAPDH

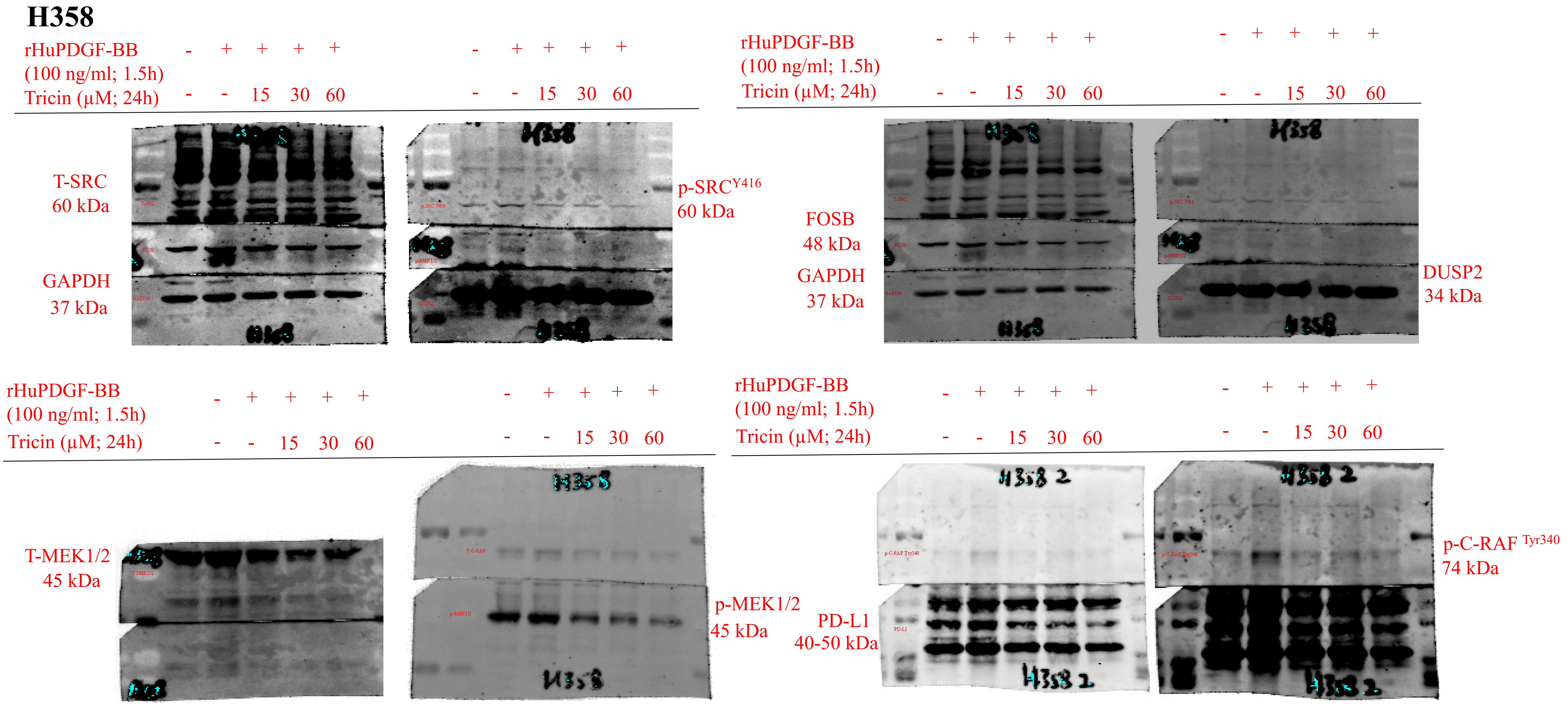
H358
rHuPDGF-BB
(100 ng/ml; 1.5h)
Tricin (µM; 24h)
- + + + +
- - 15 30 60
T-SRC
60 kDa
p-SRC Y416
60 kDa
GAPDH
37 kDa
FOSB
48 kDa
DUSP2
34 kDa
T-MEK1/2
45 kDa
p-MEK1/2
45 kDa
PD-L1
40-50 kDa
p-C-RAF Tyr340
74 kDa

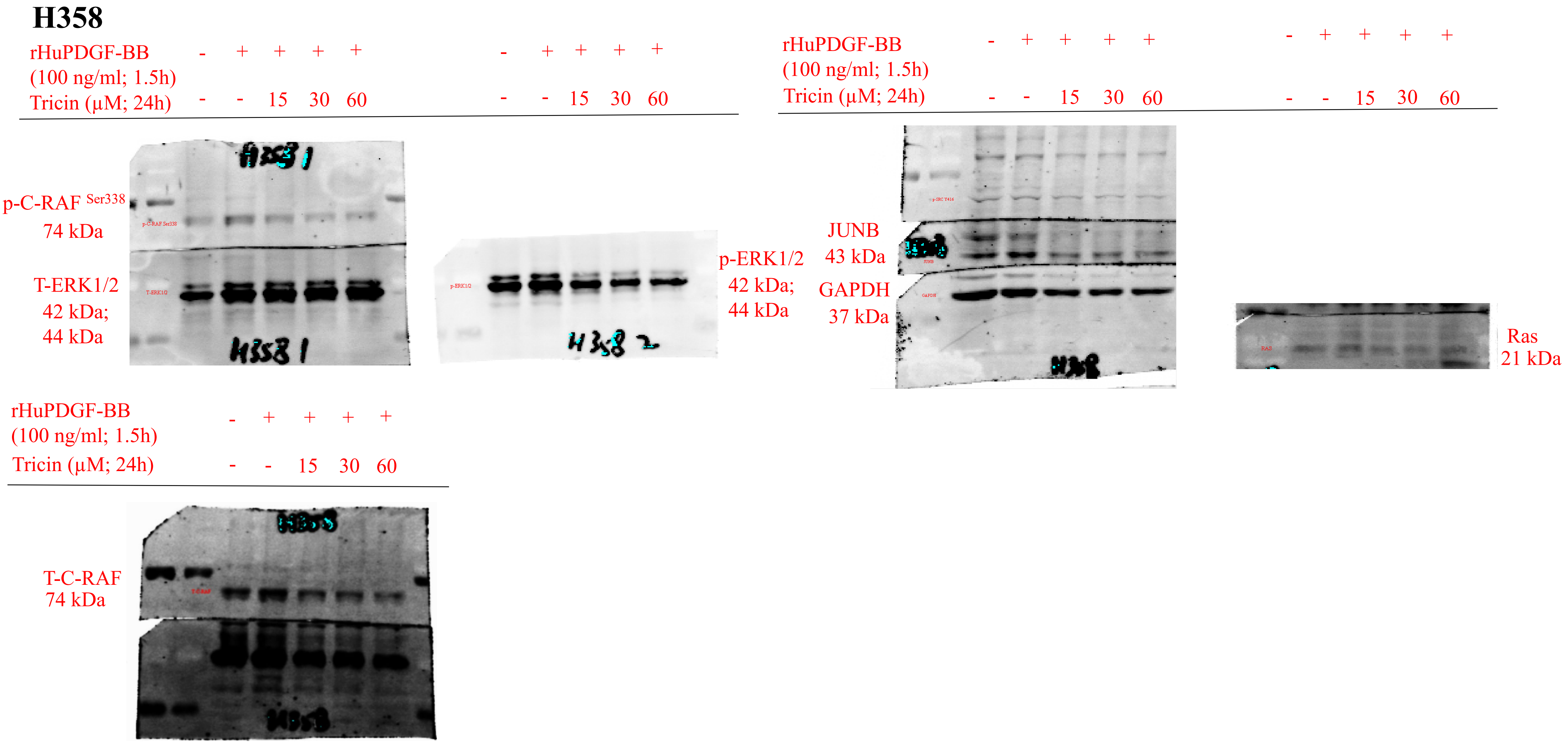

H358
rHuPDGF-BB (100 ng/ml; 1.5h) - + + + +
Tricin (μM; 24h) - - 15 30 60
p-C-RAF Ser338 74 kDa
T-ERK1/2 42 kDa; 44 kDa
p-ERK1/2 42 kDa; 44 kDa
JUNB 43 kDa
GAPDH 37 kDa
Ras 21 kDa
T-C-RAF 74 kDa

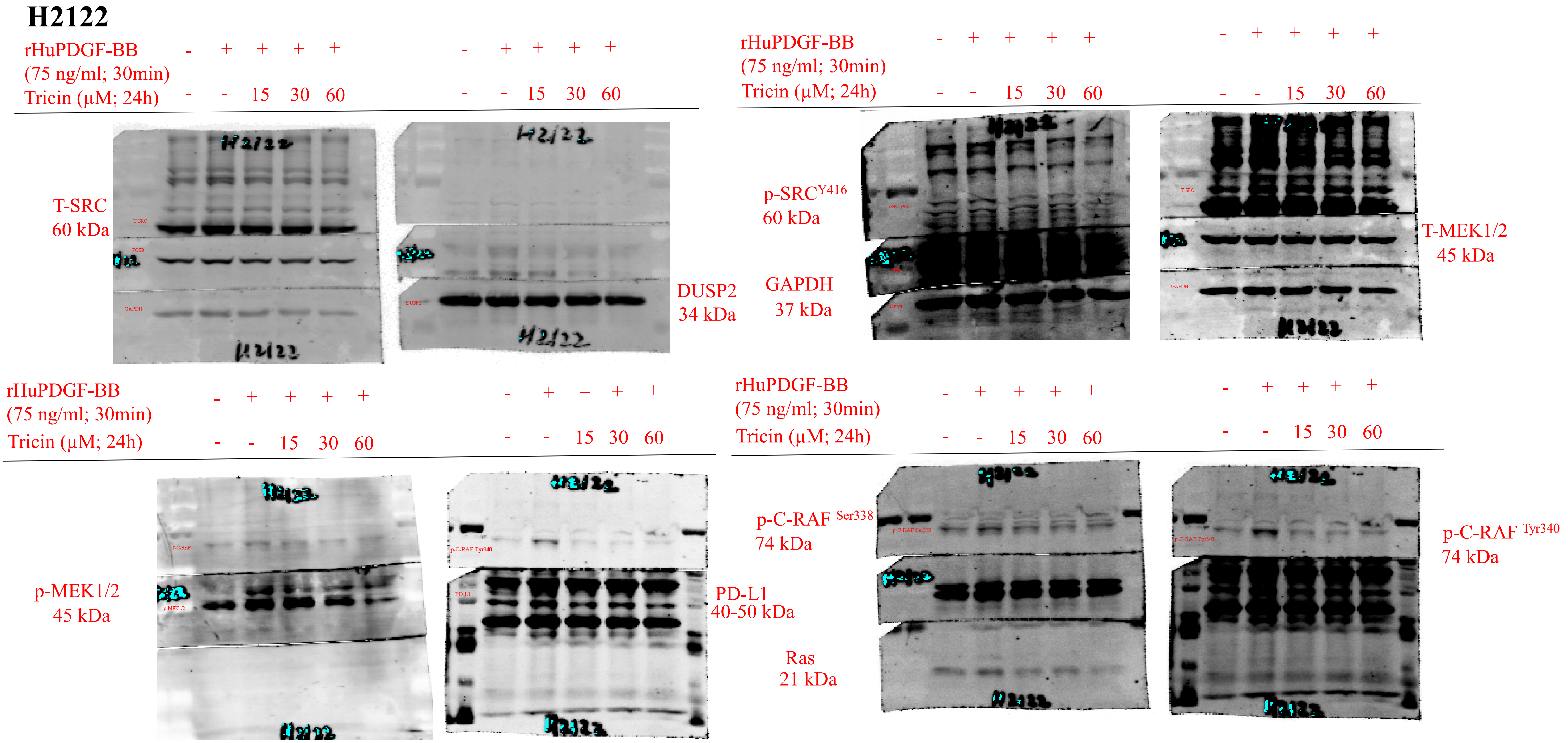
H2122
rHuPDGF-BB (75 ng/ml; 30min) - + + + +
Tricin (μM; 24h) - - 15 30 60
T-SRC 60 kDa
DUSP2 34 kDa
rHuPDGF-BB (75 ng/ml; 30min) - + + + +
Tricin (μM; 24h) - - 15 30 60
p-SRCY416 60 kDa
GAPDH 37 kDa
T-MEK1/2 45 kDa
rHuPDGF-BB (75 ng/ml; 30min) - + + + +
Tricin (μM; 24h) - - 15 30 60
p-MEK1/2 45 kDa
PD-L1 40-50 kDa
rHuPDGF-BB (75 ng/ml; 30min) - + + + +
Tricin (μM; 24h) - - 15 30 60
p-C-RAF Ser338 74 kDa
Ras 21 kDa
p-C-RAF Tyr340 74 kDa

H2122

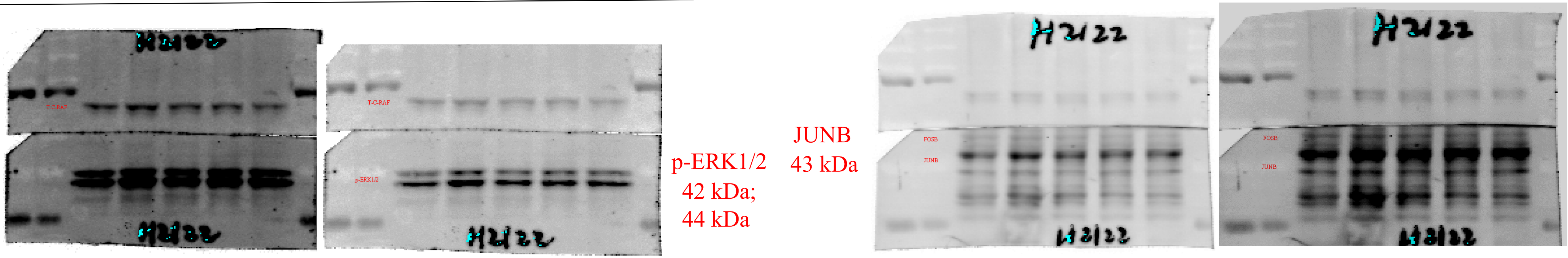

rHuPDGF-BB (75 ng/ml; 30min) - + + + + - + + + +
Tricin (µM; 24h) - - 15 30 60 - - 15 30 60
T-C-RAF 74 kDa
T-ERK1/2 42 kDa; 44 kDa
p-ERK1/2 42 kDa; 44 kDa
H2122
rHuPDGF-BB (75 ng/ml; 30min) - + + + + - + + + +
Tricin (µM; 24h) - - 15 30 60 - - 15 30 60
JUNB 43 kDa
FOSB 48 kDa

LLC

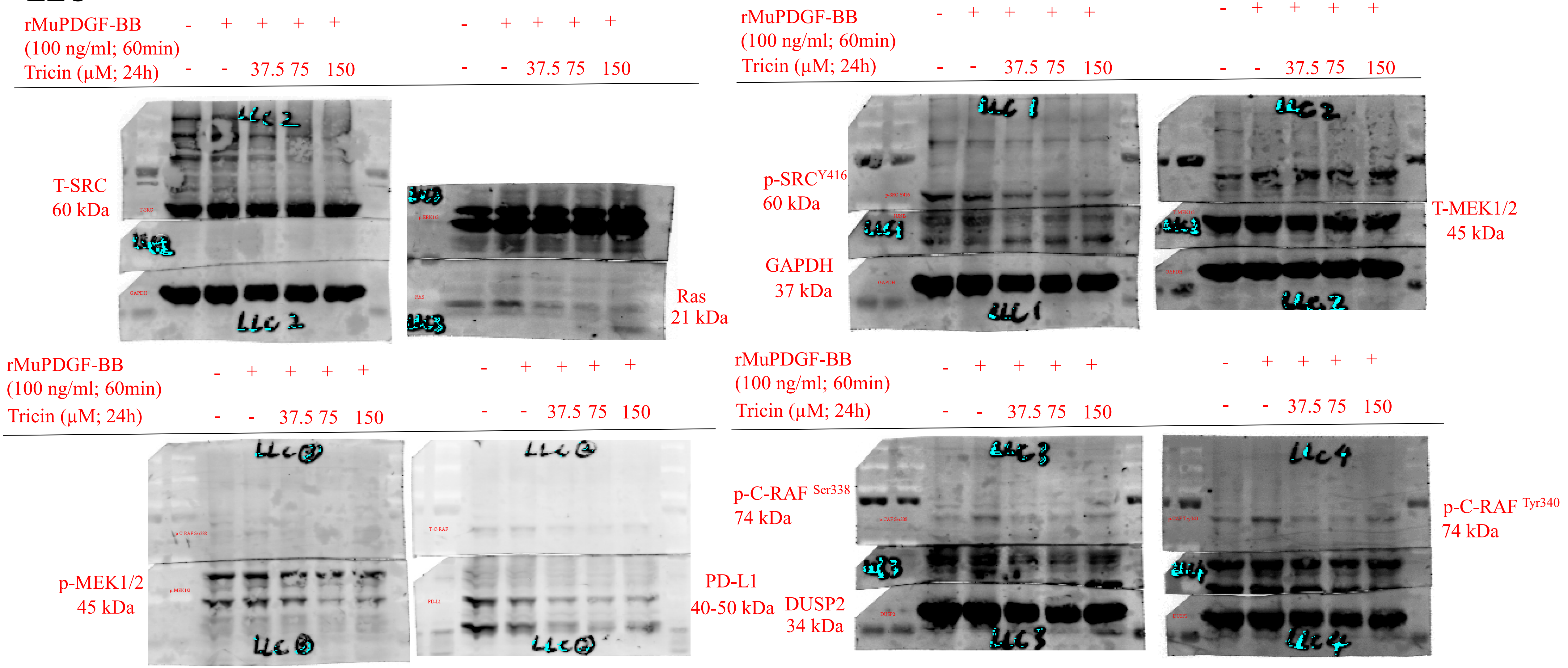
rMuPDGF-BB (100 ng/ml; 60min)
- + + + +
Tricin (μM; 24h)
- - 37.5 75 150
T-SRC 60 kDa
Ras 21 kDa
p-SRC[Y416] 60 kDa
GAPDH 37 kDa
T-MEK1/2 45 kDa
p-MEK1/2 45 kDa
PD-L1 40-50 kDa
p-C-RAF Ser338 74 kDa
DUSP2 34 kDa
p-C-RAF Tyr340 74 kDa

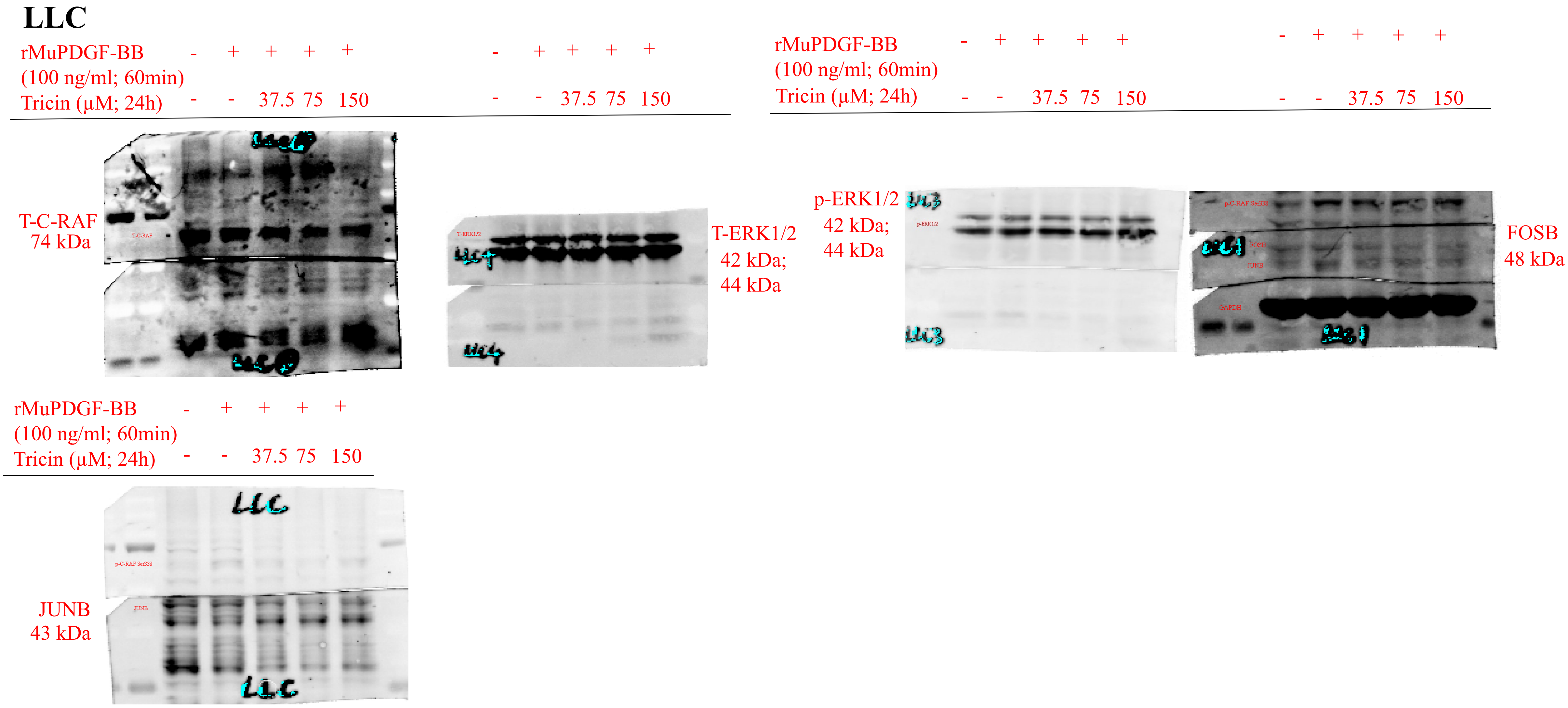
LLC
rMuPDGF-BB
(100 ng/ml; 60min)
Tricin (μM; 24h)
- + + + +
- - 37.5 75 150
- + + + +
- - 37.5 75 150
T-C-RAF
74 kDa
T-ERK1/2
42 kDa;
44 kDa
rMuPDGF-BB
(100 ng/ml; 60min)
Tricin (μM; 24h)
- + + + +
- - 37.5 75 150
- + + + +
- - 37.5 75 150
p-ERK1/2
42 kDa;
44 kDa
FOSB
48 kDa
rMuPDGF-BB
(100 ng/ml; 60min)
Tricin (μM; 24h)
- + + + +
- - 37.5 75 150
JUNB
43 kDa

H358

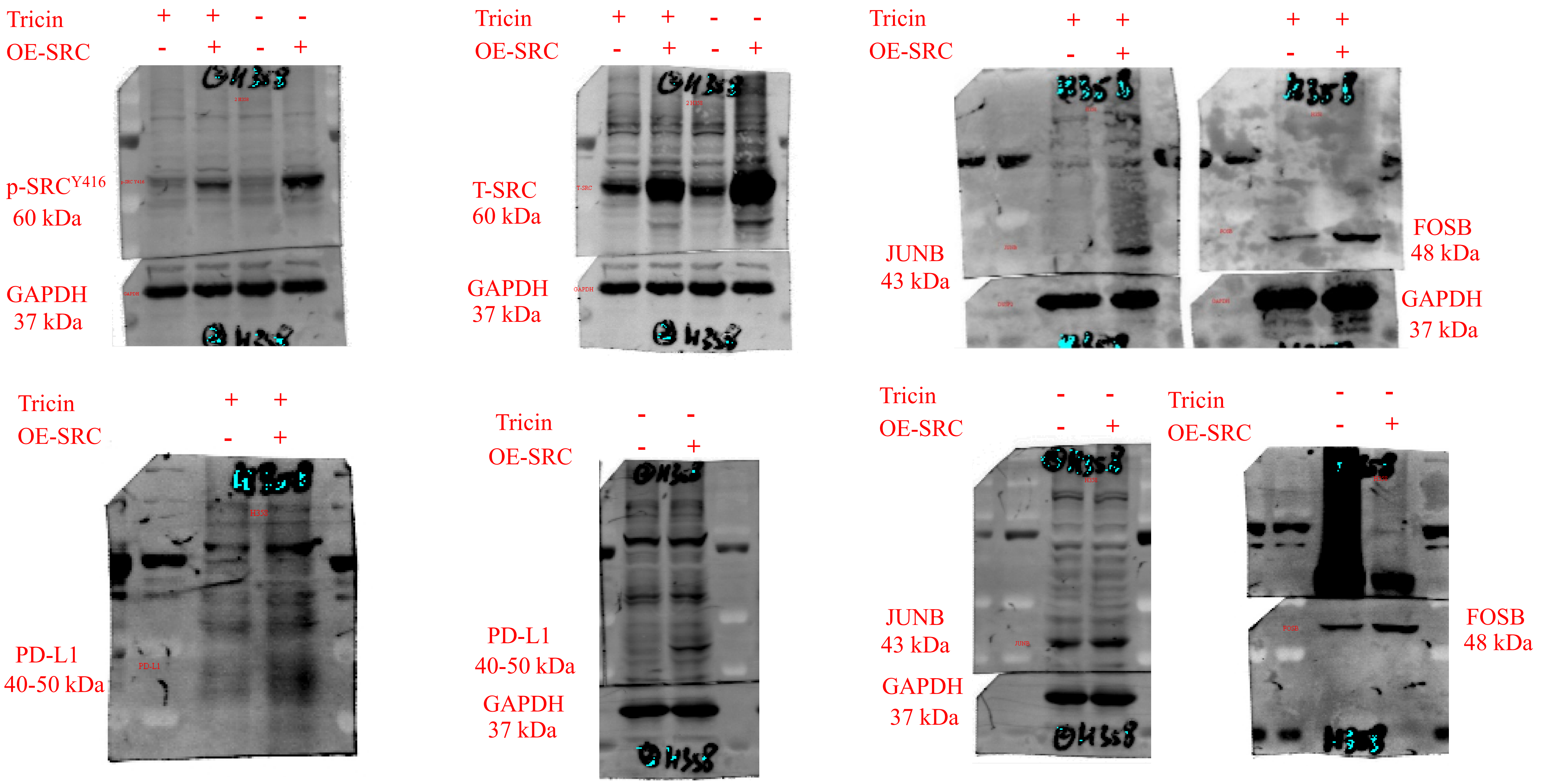
Tricin + + - -
OE-SRC - + - +
p-SRC^{Y416}
60 kDa
GAPDH
37 kDa
Tricin + + - -
OE-SRC - + - +
T-SRC
60 kDa
GAPDH
37 kDa
Tricin + +
OE-SRC - +
JUNB
43 kDa
Tricin + +
OE-SRC - +
FOSB
48 kDa
GAPDH
37 kDa
Tricin + +
OE-SRC - +
PD-L1
40-50 kDa
Tricin - -
OE-SRC - +
PD-L1
40-50 kDa
GAPDH
37 kDa
Tricin - -
OE-SRC - +
JUNB
43 kDa
GAPDH
37 kDa
Tricin - -
OE-SRC - +
FOSB
48 kDa

H2122

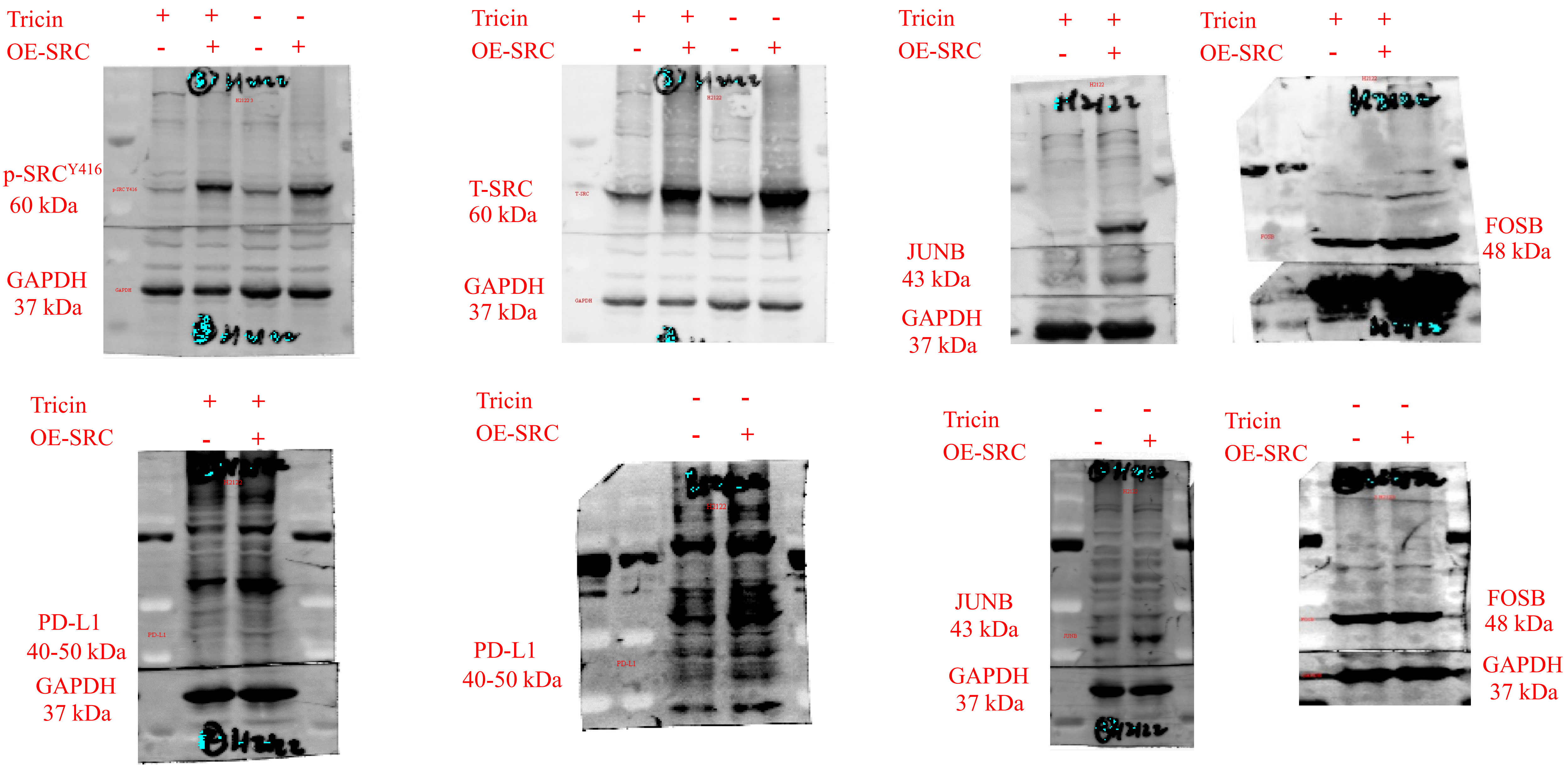
Tricin + + - -
OE-SRC - + - +
p-SRC^{Y416}
60 kDa
GAPDH
37 kDa
Tricin + + - -
OE-SRC - + - +
T-SRC
60 kDa
GAPDH
37 kDa
Tricin + +
OE-SRC - +
JUNB
43 kDa
GAPDH
37 kDa
Tricin + +
OE-SRC - +
FOSB
48 kDa
Tricin + +
OE-SRC - +
PD-L1
40-50 kDa
GAPDH
37 kDa
Tricin - -
OE-SRC - +
PD-L1
40-50 kDa
Tricin - -
OE-SRC - +
JUNB
43 kDa
GAPDH
37 kDa
Tricin - -
OE-SRC - +
FOSB
48 kDa
GAPDH
37 kDa

LLC

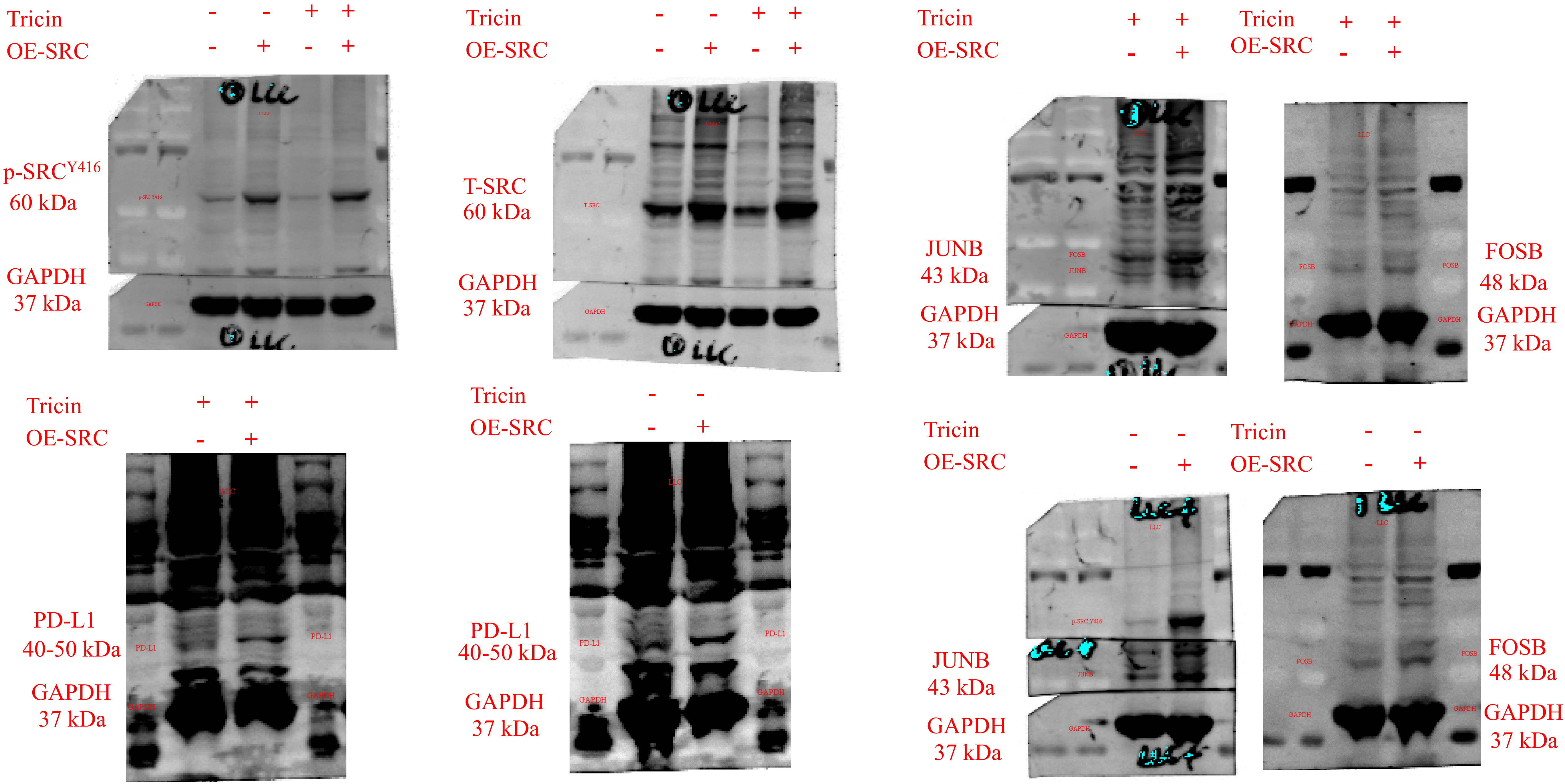
Tricin - - + +
OE-SRC - + - +
p-SRC^{Y416}
60 kDa
GAPDH
37 kDa
Tricin - - + +
OE-SRC - + - +
T-SRC
60 kDa
GAPDH
37 kDa
Tricin + +
OE-SRC - +
JUNB
43 kDa
GAPDH
37 kDa
Tricin + +
OE-SRC - +
FOSB
48 kDa
GAPDH
37 kDa
Tricin + +
OE-SRC - +
PD-L1
40-50 kDa
GAPDH
37 kDa
Tricin - -
OE-SRC - +
PD-L1
40-50 kDa
GAPDH
37 kDa
Tricin - -
OE-SRC - +
JUNB
43 kDa
GAPDH
37 kDa
Tricin - -
OE-SRC - +
FOSB
48 kDa
GAPDH
37 kDa
